# Eight-Legged Encounters—Arachnids, Volunteers, and Art help to Bridge the Gap between Informal and Formal Science Learning

**DOI:** 10.3390/insects9010027

**Published:** 2018-02-26

**Authors:** Eileen A. Hebets, Melissa Welch-Lazoritz, Pawl Tisdale, Trish Wonch Hill

**Affiliations:** 1 School of Biological Sciences, University of Nebraska-Lincoln, 402 Manter Hall, Lincoln, NE 68588, USA; 2 Department of Neurological Sciences Mind & Brain Health Labs, University of Nebraska Medical Center, Omaha, NE 68198, USA; melissa.lynn.welch@gmail.com; 3 Independent Artist, Lincoln, NE 68502, USA; gpawlt@gmail.com; 4 Methodology and Evaluation Research Core Facility, University of Nebraska-Lincoln, Lincoln, NE 68583, USA; phill3@unl.edu

**Keywords:** STEAM, evolution, biodiversity, spiders, citizen science, NGSS

## Abstract

Increased integration and synergy between formal and informal learning environments is proposed to provide multiple benefits to science learners. In an effort to better bridge these two learning contexts, we developed an educational model that employs the charismatic nature of arachnids to engage the public of all ages in science learning; learning that aligns with the Next Generation Science Standards (NGSS Disciplinary Core Ideas associated with Biodiversity and Evolution). We created, implemented, and evaluated a family-focused, interactive science event—*Eight-Legged Encounters (ELE*)—which encompasses more than twenty modular activities. Volunteers facilitated participant involvement at each activity station and original artwork scattered throughout the event was intended to attract visitors. Initial *ELE* goals were to increase interest in arachnids and science more generally, among *ELE* participants. In this study, we tested the efficacy of *ELE* in terms of (i) activity-specific visitation rates and self-reported interest levels, (ii) the self-reported efficacy of our use of volunteers and original artwork on visitor engagement, and (iii) self-reported increases in interest in both spiders and science more generally. We collected survey data across five *ELE* events at four museum and zoo sites throughout the Midwest. We found that all activities were successful at attracting visitors and capturing their interest. Both volunteers and artwork were reported to be effective at engaging visitors, though likely in different ways. Additionally, most participants reported increased interest in learning about arachnids and science. In summary, *ELE* appears effective at engaging the public and piquing their interest. Future work is now required to assess learning outcomes directly, as well as the ability for participants to transfer knowledge gain across learning environments.

## 1. Introduction

An understanding and appreciation of scientific knowledge and of science as a process is fundamental, not only to daily life, but also to the future of our planet and our societies [1]. Though an understanding of the utility of science is generally appreciated by the time students enter middle and high school, interest and engagement in science declines during this same time period [2]. Declining science interest in turn leads to declining science proficiency and persistence [3], and is expected to hinder science learning [4]. These patterns of decreased science interest and persistence result in a major societal challenge—fewer and fewer adults are scientifically literate [5,6,7]. One championed path towards increasing science literacy for youth as well as adults, is to increase the quantity and quality of science learning opportunities outside of traditional classrooms [8,9,10,11].

Though science learning can happen in a number of different of ways, can come from a variety of sources, and can happen in a multitude of environments, there are three commonly recognized sectors of education: (i) formal education in schools and universities, (ii) workplace education, and (iii) free choice learning, or informal education [12]. “Informal learning” and “informal education” were terms originally adopted by museum and environmental educators to distinguish between activities associated with in-school time versus out-of-school time reviewed in [13]. Such “out-of-school” learning can be facilitated by museums or science centers, television, radio, books or magazines, or participation in community organizations, among others. The prevalence of these free choice learning opportunities, combined with the relatively limited amount of time people spend in classrooms throughout their lives, makes informal science learning critical for the public’s understanding of science [14,15,16]. Indeed, informal science learning has been shown to account for nearly half of the public’s science understanding [15]. Given the known impact and importance of informal science education [17], it is imperative to ask how the content of informal science programming aligns, or does not align, with the science learning goals of formal science education. 

In recent years, the dichotomy between “formal” and “informal” has become less clear, and we have seen a push towards better integration, or bridging, across the two learning contexts [13,18]. Indeed, even in higher education (colleges and universities), formal learning has begun to adopt pedagogical strategies historically frequented in informal settings (e.g., *Scientific Teaching*; [19]). An ongoing transformation in undergraduate education [20,21,22,23,24], for example, is resulting in increased opportunities for students to use science process skills or to synthesize experimental results in the classroom [25]. While the edges may be blurring, clear distinctions nonetheless remain between formal and informal education—e.g., policy makers determine the goals and framework of formal, but not informal, science learning; and informal learning tends to be done on a voluntary basis, and often has a social component [13]. A movement towards the further integration of formal and informal learning is driven by the numerous proposed benefits for learners of all ages—e.g., (i) increasing motivation for learning, (ii) building “connected knowledge”, and (iii) facilitating the learning of new skills [13]. Coincident with these benefits, challenges to integration remain, and among these challenges is the alignment of knowledge content and learning goals across contexts.

The development of the K-12 Next Generation Science Standards (NGSS) [26]—formal education science standards in the United States—was guided by the U.S. National Research Council (NRC) framework, that highlights the importance of learning how to evaluate claims, how to construct arguments based on evidence, and how to interpret and understand models [27]. These standards are intended to help create a scientifically literate society. Following their implementation, it was suggested that external entities and organizations, such as scientists from higher education and research organizations, align their contributions to these new educational goals [28]. In particular, given the importance of informal science learning, an integration of NGSS has been suggested to be critical to building science learning ecosystems [28,29]; collaborative learning communities that can contribute to increased science literacy. Across the country, there are ongoing efforts to integrate across formal school curriculum and science, technology, engineering, and mathematics (STEM) learning opportunities in a variety of venues, including community science centers, museums and summer and afterschool programs [30,31]. 

In this study, we set out to contribute to the growing STEM learning ecosystem of Lincoln, Nebraska (NE), and other Midwestern communities, by using arachnids to convey science content associated with some of the biodiversity and evolution concepts in the NGSS. We did this through the creation of an interactive, hands-on informal science learning event, entitled *Eight-Legged Encounters* (*ELE*). We first implemented *ELE* at Morrill Hall, part of the University of Nebraska State Museum (UNSM), but later, broadened our reach to museums and zoos in Colorado (CO) and Ohio (OH). We focused explicitly on museums and zoos, because research has demonstrated that even single trips to a museum can have an impact on the learning of scientific concepts [32,33], including improving the conceptual understanding of evolution [34,35]. Thus, given the demonstrated importance of museum and zoo visits as vehicles for informal science learning [14,36,37,38,39], in addition to our previously established connections with such venues, we used four museums and zoos in NE, CO, and OH to test our *ELE* program.

This study leverages a unique combination of (i) personal connections between museum staff and university professors, (ii) in depth knowledge of (arachnid) organismal biology, biodiversity, and evolution, (iii) working knowledge of Next Generation Science Standards (NGSS), and (iv) a working relationship between life scientists and social scientists, to ultimately create, implement, and test an educational model that bridges informal and formal science learning through the purposeful alignment of science learning goals. We contribute directly to the field of informal science education by providing a model of collaboration among distinct sectors of education (formal and informal), and assessing its efficacy. In our evaluation of *ELE*, we explore the impact of integrating both art and social interactions (i.e., volunteers facilitating activity stations) in informal science learning experiences. Finally, we provide preliminary evidence of the usefulness of an organismal focus—in this instance, arachnids—in increasing public interest in both animals and science. In the following sections, we outline our rationale for an arachnid focus, and then provide details of *ELE* and its associated evaluation.

### 1.1. Why Arachnids?

Many invertebrates are ubiquitous, easy to maintain in a classroom, and familiar to virtually all students around the globe, making them potentially useful for engaging students in hands-on science learning. Indeed, insects and their crustacean relatives in particular have been used successfully for numerous “guided” inquiry units—e.g., pillbugs (crustacean), butterflies, mealworms, ladybugs, honeybees, mosquitoes, and termites (reviewed in [40,41]). By contrast, however, arachnids (spiders and their relatives) have not traditionally been incorporated into formal or informal science learning to the same extent (but see [42,43]). Nonetheless, like insects, many arachnids are abundant, familiar (at least spiders), easily maintained in captivity (e.g., tarantulas, [44], scorpions, [45]), morphologically and behaviorally diverse [46,47], and play important ecological roles. Spiders, for example, are estimated to consume 400–800 million tons of prey annually, making them key players in natural ecosystems [48]. The diverse and distinct properties of spider silk also make it of interest for use in a variety of biomedical applications (reviewed in [49,50,51,52,53,54]), such as bone tissue engineering [55], and as substrates to facilitate tissue regeneration [56]. Spider silk is also being used for materials innovation in areas encompassing optics, electronics, and textiles, among others [49,54]. Similarly, arachnid venoms (predominantly spiders and scorpions) are being explored for their potential use as analgesics, antimalarials, antiarrhythmics, and antimicrobials [57,58], as well as for pesticide development [59,60,61]. Ultimately, arachnids are highly relevant to our lives, ubiquitous in nature, well known, and tremendously diverse, making them well suited for engaging the public in science learning. Indeed, the extraordinary features and feats of spiders alone have been touted as a rich source of both inspiration and content for science learning across a multitude of life science core concepts [62]. Finally, arachnid morphology, behavior, and evolutionary history are distinct from those of insects, thus offering unique educational, ecological, and cultural roles in society (for an overview of insects, see [63]). 

Despite the numerous features that might make arachnids ideal organisms to leverage for hands-on science learning, students and teachers alike are often afraid of and/or dislike arachnids. Children and adults, for example, tend to have negative attitudes towards spiders ([64], reviewed in [65]). We propose that such negative attitudes, however, are likely associated with a lack of knowledge. Deficient knowledge of arachnids among the general public is demonstrated by the fact that children often categorize spiders with insects, despite their distinct form and evolutionary history [66]. Even adults group spiders with the most “disliked” bugs [67]. Among young adults, for example, 27% of surveyed U.S. college students included “spiders” in their list of disliked bugs [67]. Prior research using live spiders with children in an educational setting, however, found that arachnid interventions could lead to decreased fear and disgust of spiders, as well as increased positive environmental beliefs [42]. A more recent study incorporated exemplars from five living arachnid orders to explore human attitudes towards arachnids and similarly found that arachnid interventions can lead to decreased fear, perceived danger, and disgust [43]. This prior work demonstrates that negative attitudes towards arachnids are not barriers to their inclusion in science education. Notably, spiders were also the most commonly recalled “bug” [67] among adults; meaning that spiders are highly memorable. Indeed, more than twenty years of research and public engagement using arachnids (Hebets, EA personal observation) suggests that people of all ages are simultaneously excited, worried, intrigued, scared, and captivated by arachnids. We suggest that the strong emotional response elicited by arachnids can provide a foundation and entry point for stimulating science learning. 

### 1.2. ELE Overview and Goals

*Eight-Legged Encounters* (*ELE*) consists of a series of interactive modular science activities that use arachnids to convey life science ideas aligned with the NGSS core concepts of Biodiversity and Evolution. Overarching goals of *ELE* were to increase science interest, knowledge, understanding, and future science engagement in event participants—especially knowledge and understanding related to the focal NGSS core concepts. By bridging informal learning experiences with formal learning goals, we aimed to provide both a foundation of knowledge and an additional context of knowledge upon which formal education can build. Immediate goals of *ELE* were to engage visitors; increase positive attitudes towards arachnids, explicitly spiders; and increase interest in science. While likely impactful on its own, *ELE* was meant to contribute yet another dimension to local STEM learning ecosystems.

In terms of evaluation, we focused explicitly on questions regarding (I) activity-specific visitor interest; (II) the impact of art and volunteers on overall visitor engagement; (III) improved attitudes towards spiders; and (IV) increased interest in science (Table 1). As this study encompasses the initial development and efficacy-testing of *ELE*, assessments of knowledge gain were beyond the scope of our current evaluation. This current study focuses on the efficacy of the overall model, but does not yet explore the long term impact of the model on the participants and/or the community.

The primary audience for *Eight-Legged Encounters* was visitors to the museum/zoo on the day of the exhibit. The *ELE* event at the University of Nebraska State Museum’s Morrill Hall (hereafter UNSM) in spring 2013 was the inaugural event. In fall 2013, a subset of *ELE* activities was presented at the Butterfly Pavilion in Chesterfield, CO (BP). In spring 2014, UNSM hosted the second *ELE* (UNSM 2014), followed by another event in fall 2014, hosted by the Denver Museum of Nature and Science (DMNS) in Denver, CO. Finally, the Toledo Zoo hosted a subset of *ELE* activities during their annual “BugFest” in fall 2017 (TZ). The two *ELE* events at UNSM were well advertised, and many visitors came to the museum explicitly for the exhibit. In contrast, *ELE* was not well advertised at the other sites, and thus, the majority of visitors attending the museum/zoo were not there with the explicit intent of participating in *ELE* activities. 

*Eight-Legged Encounters* used two primary means of engaging visitors—(a) live volunteers that engaged with participants at each activity station, and (b) original artwork in various forms throughout the exhibit (detailed in *ELE Activities*). We explicitly used volunteers because “interactivity” and informal, interpersonal social interactions are suggested to be critical to learning and engagement [68,69]. In terms of original artwork, we recruited and collaborated with a local artist (P.T.) so as to facilitate a non-scientist’s interpretation of arachnids.

Each *ELE* station typically had 1–3 volunteers at all times, depending upon the size of the venue and the number of available volunteers. Many of the volunteers were graduate students from the University of Nebraska-Lincoln (UNL, all five events), the University of Colorado Boulder (BP), the University of Colorado Denver (BP), and Bowling Green State University (TZ). Additional volunteers included staff at local zoos (e.g., Henry-Doorly Zoo, UNSM), and docents and volunteers from the DMNS. Undergraduate students, as well as fellow faculty from UNL (UNSM; DMNS) and Bowling Green State University (TZ), also participated as volunteers. The level of science background across volunteers was variable. Undergraduates were predominantly Biology majors, graduate students were mostly from Ecology, Evolution, and Behavior oriented programs, and faculty were mostly from Biology and Psychology departments. There were no incentives for volunteers (e.g., no extra credit) other than receiving an *Eight-Legged Encounters* tee-shirt. Volunteers were recruited predominantly through personal inquiries and email listservs.

Volunteers were assigned a station(s) prior to the event, and were provided with volunteer “cheat sheets”, which they were asked to look over and study. In most cases, previously trained volunteers were able to run through mock demonstrations with all new volunteers prior to their participation.

The event explicitly targeted families by purposefully incorporating activities that we imagined would be engaging for a range of ages—from toddlers (e.g., Read Aloud) to adults (e.g., Community Experiment). Our expectation was that if we provided engaging activities for a wide range of audience ages, entire families would be more likely to come, would stay longer, would learn more, and would be more engaged overall. Parents and other family members are known, for example, to have strong influences on student attitudes about pursuing science [70,71,72]. Shared family experiences in informal science learning has also been shown to increase both science interest and science self-efficacy ([73,74], reviewed in [75]).

### 1.3. ELE Conceptual Themes

When designing *Eight-Legged Encounters* activities, we focused on two broad conceptual themes: Biodiversity and Evolution. Biodiversity knowledge, especially among youth, is frequently biased towards vertebrates—e.g., mammals, birds, and reptiles. Ironically, the taxa that represent the majority of earth’s biomass and species richness—i.e., arthropods—are underrepresented [76]. Indeed, there are far more species of spiders (47,099; [77]) than there are mammals (<5500), birds (~10,000), and reptiles (lizards, snakes, turtles, crocodiles, tuataras, and amphisbaenians) (10,450; [78]) together. Biodiversity, and conserving the earth’s biodiversity, is imperative for the health of our planet, as well as ourselves [79], and we attempted to use arachnids to convey some of this amazing biodiversity. Importantly, biodiversity is a Disciplinary Core Idea in the NGSS (*LS4.D: Biodiversity and Humans*; see Table 2, Table 3, Table 4 and Table 5).

Coincident with conserving earth’s biodiversity is a necessary understanding of evolution. Indeed, understanding evolution is relevant for conservation, agriculture, and medicine—it is imperative for helping us solve our most pressing local and global challenges [80,81,82]. Unfortunately, the United States ranks second to last among the industrialized nations in terms of acceptance of evolution [83], and a number of studies demonstrate that even those that accept evolution demonstrate fundamental misunderstandings (reviewed in [84]). Given these deficiencies, formal education appears inadequate in providing a foundation of understanding of evolutionary theory [84]. As a result, many museums across the world have begun to develop and evaluate exhibits targeting evolutionary theory, focusing their evaluation on the effectiveness of such exhibits in contributing to conceptual change with respects to reasoning about evolution [34,35]. Given this, we leveraged arachnids to convey Disciplinary Core Ideas in the NGSS associated with evolution (*LS1.A: Structure and Function*; *LS3.B: Variation of Traits*; *LS4.A: Evidence of Common Ancestry and Diversity*; *LS4.B: Natural Selection*; and *LS4.C: Adaptation*; see Table 2, Table 3, Table 4 and Table 5).

In addition to the specific Biodiversity and Evolution-based core ideas, we additionally incorporated the following core concepts into various activities: *LS1.D: Information Processing*; *ETS1.C: Optimizing the Design Solution*; *LS1.B: Growth and Development*; and *LS2.A: Interdependent Relationships in Ecosystems* (see Table 2, Table 3, Table 4 and Table 5). We also incorporated the Science and Engineering Practices of *Developing and Using Models* and *Asking Questions and Defining Problems*, as well as the cross-cutting concepts of *Structure and Function*; *Scale*, *Proportion*, and *Quantity*, and *Science Addresses Questions about the Natural and Material World* (see Table 2, Table 3, Table 4 and Table 5).

### 1.4. ELE Activities

With the themes of Biodiversity and Evolution in mind, we identified four distinct categories of activities: (I) *Classification and Taxonomy*, (II) *Spiders and Silk*, (III) *Path of Predators*, and (IV) *Hands-on Science*. Throughout, we tried to incorporate best practices in informal science education, for example, using live animals when possible [85]. We outline the associated activities below.

#### 1.4.1. Classification and Taxonomy

*Classification and Taxonomy* activities included any number of the following discrete modules: *What is an Arthropod*, *Create a Chelicerate*, and *Assemble an Arachnid* (Table 2). These modules all focus on the arachnids and their arthropod relatives—i.e., what physical characteristics unite and/or distinguish different groups of arthropods, and to what animals are arachnids most closely related? What is their evolutionary history and their evolutionary relationships? Activities in this general category include games, as well as arts and crafts.

#### 1.4.2. Spiders and Silk

The *Spider and Silk* activities include of any number of the following discrete modules: *Build a Burrow*, *Catch a Moth*, *Sticky vs. Wooly Silk*, *Tissue Paper Flower*, *Weave a Web*, *Sound Station*, and *Read Aloud*. Many of the modules within this category focus on one extraordinary feature of spiders—their ability to produce multiple kinds of silk which they use in a diversity of distinct ways (e.g., protecting eggs, building shelter, catching prey, wrapping prey, transferring sperm, etc.). Similar to the Classification and Taxonomy stations, these activities also include games, arts and crafts, as well as free play (Table 3).

#### 1.4.3. Path of Predators

The *Path of Predators* is a set of eleven stand-alone stations representing the eleven living arachnid orders—Acari, Amblypygi, Araneae, Opiliones, Palpigradi, Pseudoscorpiones, Ricinulei, Schizomida, Scorpiones, Solifugae, and Thelyphonida (Table 4). Though they were originally created to work together in their entirety, the *Path of Predators* can be trimmed down to any size, depending upon the availability of space and staff. For example, while the *ELE* events in NE and CO included the full eleven stations, space limitations required us to modify the full exhibit into a reduced form at the Toledo Zoo, OH. Our modified *Mini Path of Predators* included Amblypygi, Araneae, Scorpiones, and Thelyphonida.

**Figure 1:**
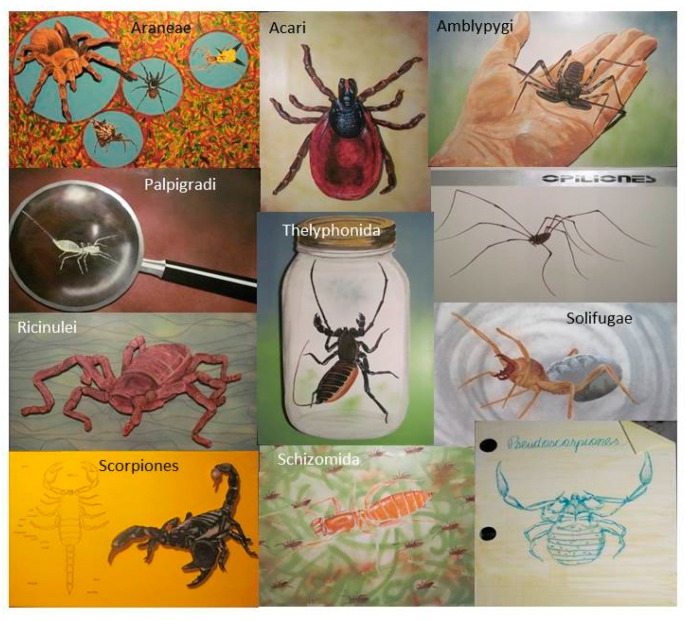
Original artwork painted onto foam poster boards (3 × 5 ft) and held on easels attracted participants to the eleven biodiversity stations associated with the *Path of Predators*.

**Figure 2:**
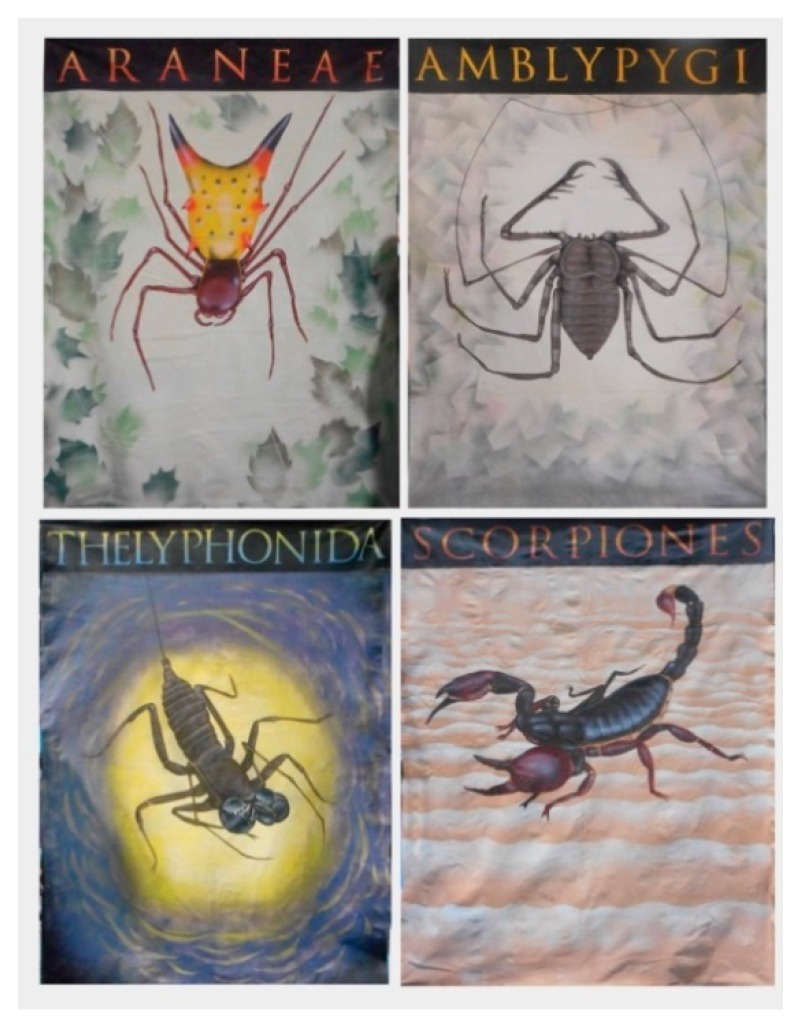
Six-by-eight foot original canvases were used to draw in attendees at the Toledo Zoo’s *Path of Predators* event.

**Figure 3:**
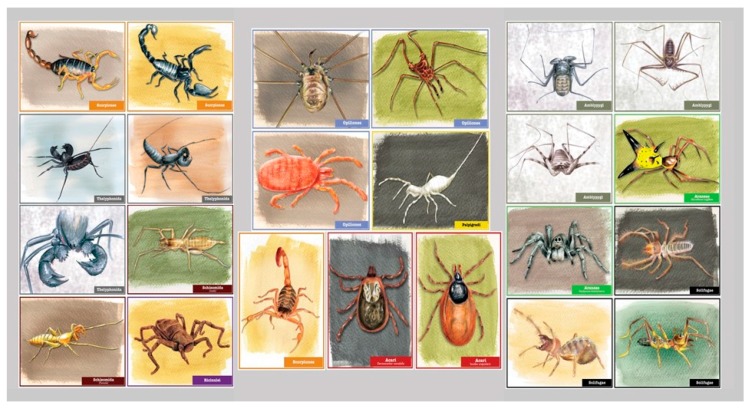
Original artwork trading cards were handed out to youth following the completion of the *Path of Predators* activities. Youth were able to choose one of two or three different designs. The backs of each card had information regarding the following topics: Order name; Common Name (when available); Habitat; and Fun Fact.

The *Path of Predators* and the *Mini Path of Predators* have associated activity booklets that go along with the stations. The booklets invite participants to grab a pencil, go on a journey through the exhibit, and discover the wonderful world of arachnids (Supplementary Materials—*Path of Predators* and *Mini Path of Predators*). The accordion-style booklets have one page dedicated to each arachnid order. These pages provide instructions for participants, and often provide opportunities for writing and/or drawing. Each page also has an “At Home” section, with suggestions of activities that can be done outside of the event. The first page of the booklet additionally has a phylogenetic tree representing the hypothesized relationships among the eleven orders. As participants complete an activity at each order station, volunteers stamp their activity booklet in the order-specific location on the phylogenetic tree. Upon completion of all activities and receipt of all stamps, participants can show their completed booklet to a volunteer at the front table and they receive a prize (i.e., toy spiders initially, and then original artwork tattoos). Each *Path of Predator* station was composed of 1–2 tables covered with photographs, preserved specimens, and when possible, live specimens in transparent containers. In particular, ELE often had live whip spiders (Amblypygi), spiders (Araneae), harvestmen (Opiliones), pseudoscorpions, scorpions (Scorpiones), and vinegaroons (Thelyphonida). Each *Path of Predators* station additionally had its own original artwork sign—3 × 5 ft foam poster board (Figure 1), except at the *Toledo Zoo*, which had 6 × 8 ft original artwork painted canvases (Figure 2). Each *Path of Predator* station also had an original stamp (Supplementary Materials—Figure S1), a series of two to three trading cards with original artwork on one side and fun facts on the other (Figure 3), a set of original sculptures (Supplementary Materials—Figure S2), and at least one interactive activity (Table 4).

#### 1.4.4. Hands-on Science

The *Hands-on Science* activities included two modules that allowed participants to play the role of scientists (Table 5). In particular, the *Community Experiment* allowed participants to collect their own live spider, run it through a pre-designed experiment, collect data, enter data, observe real-time data trends, and ultimately read a final write-up of the experiment. Due to space and logistical constraints, the *Community Experiment* only took place during the UNSM events in Lincoln, NE.

A short video of *ELE* in action at the Denver Museum of Nature and Science can be found online [87].

#### 1.4.5. Sample Activity—Community Experiment

The following scenario walks you through a scene for the Community Experiment. Note that this experiment involves live spiders and live crickets. Spiders were relatively small ground dwelling wolf spiders, *Schizocosa floridana*, that were collected by members of the Hebets’ Laboratory near Gainesville, FL. 2013 and 2014. Approximately 400 spiders were collected for each Community Experiment, and crickets were ordered from Ghann’s cricket farm (GA, USA).

VOLUNTEER—standing near a door. (Family walking past) *Would you like to come help us out with an experiment we’re conducting?* (Ideal answer: “sure”. If the answer is no, volunteer encourages participation by assuring them it won’t take too long. Volunteer tells them they will be able to collect their own spider as well as their own data, etc.)

*The first thing I would like you all to do is to put on a headlamp, turn it on, and follow me. We are going to enter a completely dark room and I’m going to ask you to look around and tell me what you see.* (Enter darkened room. Participants report seeing large plastic tubs on the floor filled with dirt and some moss or leaves.)

*Can you see anything IN the tubs? In particular, do you see any color?* (Participants report seeing small green shimmers—these are live spiders!) 

*What do you think that is? Does it remind you of anything?* (Following some further inquiry and discussion, the volunteer reveals that the green shimmers are light reflecting off of spider’s eyes. The volunteer briefly describes how spiders have a tapetum lucidum, just like cats and dogs, and that this light-reflecting layer at the back of the eye helps nocturnal animals acquire more light so that they can see better at night.)

*Would you like to catch one of these spiders to use in our experiment?* [Volunteer guides the participant to a bin and demonstrates how to catch a spider. S/he then allows them to catch their own live spider.] 

Now let’s go test your spider!

WHAT HAPPENS NEXT: The collecting vial provided to capture the spider has a pre-assigned colored dot on it, indicating the experimental treatment: blue = rock, green = paper. The family is guided to an experiment station matching their vial’s color (blue vs. green). The volunteer engages them in a discussion about how spiders acquire information about the world around them. *Do spiders have eyes?* (yes, most do) *How many?* (most have 8) *Noses? Can spiders smell?* (no, they don’t have noses. They do, however, have hairs that can detect chemicals) *Do spiders have ears?* (no, not ears like us. They have slits in their exoskeleton that can detect vibrations and they have hairs that can detect the movement of air particles) *What senses do you think are most useful for spiders in detecting and capturing prey?* (probably vibration) *This experiment tests the hypothesis that spiders rely on vibrations for prey capture. It tests that hypothesis by comparing the time it takes spiders to catch prey on a surface that does versus does not transmit vibrations (paper vs. rock, respectively)*.

**Figure 4:**
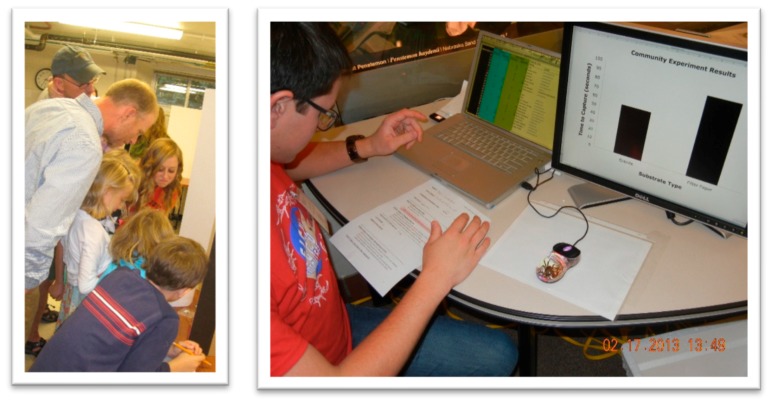
Families gather around an arena to watch and collect data on a spider attacking a cricket in the *Community Experiment* (LEFT photo) and then enter their data into the computer and view the results of the experiment in real-time (RIGHT photo).

Each experimental station is equipped with: a plastic arena with a bottom of either (a) rock or (b) paper; a stopwatch; a data sheet and pencil; and a container of live crickets and soft forceps. The family ultimately conducts a foraging experiment, whereby they place their spider in the arena for a set period of time. They then catch a cricket, place it at the opposite end of the arena as the spider, start their stopwatch, and observe the spider forage (Figure 4)! They write their data down on their data sheet and then proceed to the computer station where an excel file is already open and ready for their data entry (Figure 4). A second monitor shows a graph that changes in real-time as new data are entered. Completed write-ups of the experiments can be found online (see RESULTS February 2013 and September 2013, [88]).

Volunteers remained nearby to answer any questions and/or help in any way. Common challenges involved: spiders that would not eat; spiders that ate before the stopwatch began; participant difficulty catching crickets; crickets escaping. In all cases, the volunteers discussed various options to overcoming the challenge(s) with the family, and together came up with a solution. If a spider did not eat within a set amount of time, for example, (typically 3–5 minutes depending on the patience level of the family) sometimes a family member would return to the darkened room to collect another spider.

## 2. Materials and Methods

The data for this paper comes from a multi-year evaluation of the one-day, informal family-based science learning event entitled *Eight-Legged Encounters*. The evaluation data was collected by the Bureau of Sociological Research (BOSR) at the University of Nebraska-Lincoln (UNL), between September 2013 and September 2014, and again, in September 2017. The evaluation data include data collected from seven different surveys (Supplementary Materials—Surveys a-g) across five distinct *ELE* events at four different venues (Table 6). 

### 2.1. Adult/Family Participant Survey

Using self-reflective surveys, respondents were asked a series of questions about their interest and engagement with different *ELE* activities, and about how effective the volunteers and the artwork were in the *ELE* exhibits (see Supplementary Materials for all surveys, a-g). Participants were also asked how their participation affected their attitudes and interest in learning more about spiders, and about science more generally, and if applicable, whether it made them more likely to consider a future job in science. They were asked whether they learned anything new, and whether they thought it was important for these kinds of activities to be available to the public. 

Surveys were developed by BOSR staff in consultation with EAH. Slightly different surveys were used for Adults/Families across our events (see Table 6). Distinct surveys were due, in part, to inconsistency in the evaluators throughout the study. We note that M.W.L. and T.W.H. provided analyses of already collected data, and were not part of the BOSR team that built or administered the survey tools. Additionally, activities were iteratively revised following formative feedback, and as such, the team was interested in different aspects of *ELE* (e.g., different activities) across events. Nonetheless, despite slight differences in surveys, numerous questions were overlapping, thus enabling us to address our focal evaluation questions (Table 1). The total number of adult/family participants surveyed across four events reported here was three hundred and fifty. Gender and age were not collected as part of the adult/family surveys.

### 2.2. Youth Participant Survey

During two of the four events (UNSM 2014 and DMNS), youth were also surveyed (Supplementary Materials, Survey f—UNSM 2014 and Survey g—DMNS Youth Survey). Youth surveys at UNSM 2014 were administered by a BOSR staff member. In instances where youth could not complete their surveys alone, youth survey completion at DMNS was facilitated by parents. The age range of youth respondents was wide, from preschool age to late teens (average age was 8.5 years). Because of this, the youth evaluation survey was short and simple. Youth were asked if they want to learn more about spiders, if they want to learn more science, and if they want a job in science. The total number of youth surveyed was 165 across two separate events with a 49:51 percent boy/girl ratio. Unfortunately, we did not yet have a youth survey developed for the UNSM 2013 event, and we did not have enough volunteers to administer youth surveys at the BP or TZ.

### 2.3. ELE Activity Interest

**Figure 5:**
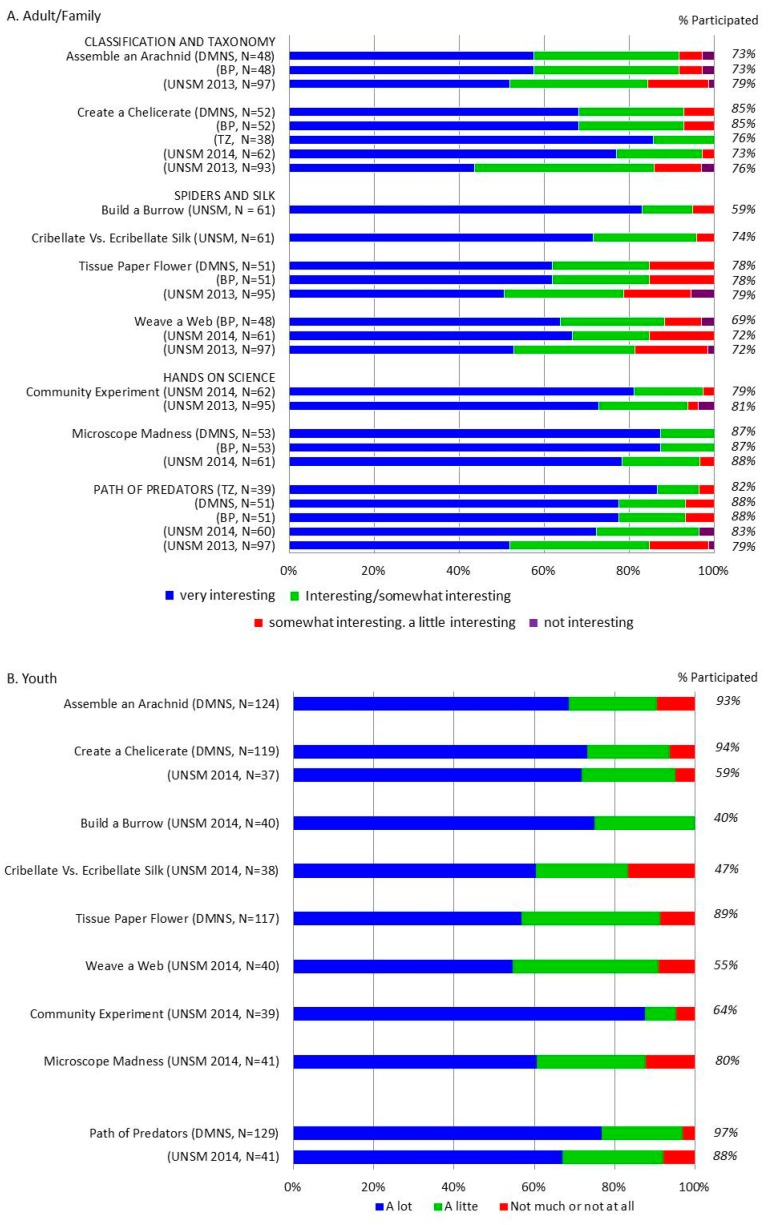
The majority of adults/families (**A**) participated in the surveyed stations, and the majority found them to be “very interesting” or “interesting/somewhat interesting”. The youth (**B**) reported lower participation at some surveyed stations (e.g., Cribellate vs. Ecribellate Silk and Build a Burrow) but similar to adults, the majority of surveyed youth found the activities to be “a lot” or “a little” interesting. Across both groups, there was only a small number of participants reporting “not interesting” (4% in adults; 10% in youth).

Nine specific activity stations were included in this evaluation across one or more *ELE* events: *Assemble an Arachnid*, *Create a Chelicerate*, *Build a Burrow*, *Cribellate vs. Ecribellate Silk (i.e., Sticky vs. Wooly)*, *Tissue Paper Flower*, *Weave a Web*, *Community Experiment*, *Microscope Madness*, and *Path of Predators* (see superscript codes in Table 3 and Table 5 for venue-specific activities). Due to (a) site-specific space constraints, (b) limited numbers of volunteers, and/or (c) revisions based on formative feedback, *ELE* did not always have the same combination of activities. As such, the inclusion/exclusion of specific activities differs across events. Importantly, the potential responses available to participants also differed across surveys. For example, in the UNSM 2013 survey, the two most positive responses were “very interesting” and “interesting”, while in the UNSM 2014 survey they were “very interesting” and “somewhat interesting”. Nonetheless, across all surveys, there were four potential responses with “very interesting” and “not interesting” acting as bookends. As such, in our graphical representations, we combined responses from the most positive choices (“very interesting”), the second most positive choices (“interesting” or “somewhat interesting”), the third most positive choice (“somewhat interesting” or “a little interesting”), and the least positive choice (“not interesting”) across surveys (see legend, Figure 5). The remaining data on engagement and interest include data from all of the events, as summarized in Table 6.

### 2.4. Additional Data

Volunteers were also surveyed at two of the four events (UNSM and DMNS; see Supplementary Material—Volunteer Survey). Volunteers were asked about whether volunteering in the event made them more or less likely to volunteer again in the future, to want a job in science or education, or if it had an impact in their interest in communicating science to the public. The total number of volunteers surveyed was 87 across two separate events. Roughly 38% of volunteers were under the age of 25, and another 40% were between 25 and 34 years old. Fifty-eight percent of the volunteers were women.

## 3. Results

*Eight-Legged Encounters* attracted ~847 visitors at the UNSM in 2013 and ~816 at the UNSM in 2014. A total of ~500–600 visitors participated in *ELE* at the BP; >3000 at the DMNS; and >2,500 participants at the TZ. Again, most of the participants at the UNSM 2013, 2014 came explicitly for the exhibit, while the other venues (BP, DMNS, and TZ) took advantage of visitors present for normal museum/zoo visits. It is notable that the DMNS *ELE* took place on their community free day.

### 3.1. ELE Activities—Visitation and Interest

Across all sites (UNSM, BP, DMNS, and BP), we found similar activity-specific participation for adults/families. Of the activities surveyed, *Microscope Madness* and the *Path of Predators* tended to be visited most frequently: between 79% and 88% of adults/families reported visiting and participating in these stations (Figure 5A). All activities were visited by the majority of participants, with the lowest visitation rate being 59% (*Build a Burrow*). Self-reported visitation or participation rates were similar across the youth surveys. For youth, *Microscope Madness* (80% participated) and the *Path of Predators* (88% and 97% participated across UNSM 2014 and DMNS respectively) had the highest participation (Figure 5B). Youth also reported high participation in *Assemble an Arachnid* (93% at DMNS) and *Create a Chelicerate* (94% at DMNS but only 59% at UNSM 2014). 

Adults/families found all of the activities interesting. When we look at the combined results from the top two interest categories for adults/families (i.e., “very interesting” and “interesting/somewhat interesting”), the most interesting activities were reported to be *Microscope Madness* (85–87%), the *Path of Predators* (67% at UNSM 2013 and 80%–83% across all other venues), and the *Community Experiment* (76–78%) (Figure 5A). The *Weave a Web* station (UNSM 2013) and *Tissue Paper Flower* (UNSM 2013) received the lowest interest scores, with 59% (*Weave a Web* UNSM 2013) and 62% (*Tissue Paper Flower* UNSM 2013) of participants reporting finding the activities “very interesting” or “interesting/somewhat interesting”. The interest scores for both of these stations increased slightly following revisions. At the most recent *ELE* in which they were both incorporated, *Weave a Web* received a combined “very interesting” and “interesting/somewhat interesting” score of 61% (BP), while *Tissue Paper Flower* (BP and DMNS) received a 67%.

Among the youth, the *Path of Predators* (81–94%), *Create a Chelicerate* (88%), and *Assemble an Arachnid* (84%) received the highest combined scores of “a lot” and “a little” when youth were asked how interesting they found activities (Figure 5B). Two activities received youth interest scores below 50%: *Cribellate vs. Ecribellate Silk* and *Build a Burrow* (both 40% combined interest score). Interestingly, these scores were much lower than those reported in the adult/family surveys at the same events (adult/family scores: 71% for *Cribellate vs. Ecribellate Silk* and 56% for *Build a Burrow*). 

In summary, all activity stations appeared to capture the interest of adult attendees—only 4% or fewer rated any of the stations as “not interesting” (*Tissue Paper Flower*, UNSM 2013, Figure 5A). For youth, 10% of those surveyed found *Microscope Madness* “not much or not at all” interesting. The *Path of Predators* (DMNS), the *Community Experiment* (UNSM 2014) and *Create a Chelicerate* (UNSM 2014) all scored <5% for “not much or not at all” interesting, and the remaining activities received scores between 5–9% (Figure 5B). 

### 3.2. ELE Engagement—Volunteers and Art

**Figure 6:**
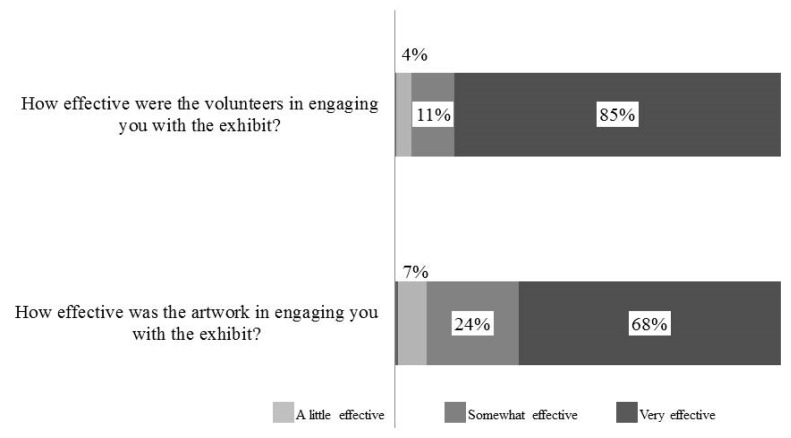
The majority of participants reported that the volunteers (top bar) and the artwork (bottom bar) were “very effective” in engaging them with the exhibits. Data included are from BP, UNSM 2014, DMNS, and TZ.

*Eight-Legged Encounters* events utilized two main strategies for visitor engagement—volunteers and artwork. Attendees from all five events (UNSM 2013, 2014; BP; DMNS TZ) reported volunteers to be the most effective engagement strategy—85% said volunteers were “very effective” and 11% said volunteers were “somewhat effective” (Figure 6). More than two-thirds of adult attendees (68%) reported artwork was a “very effective” engagement strategy, and 24% said it was “somewhat effective” (Figure 6).

### 3.3. ELE Adult/Family and Youth Impact—Spiders and Science

**Figure 7:**
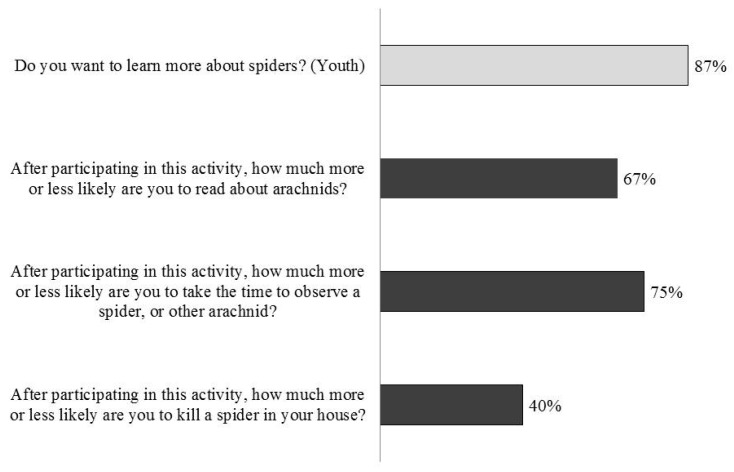
The majority of adult and youth participants reported increased interest in spiders or to relevant questions. The youth question (top) shows the proportion of youth responding “yes”. Adult questions show the combined adult responses from “much more likely” and “more likely”, except in the case of the “kill a spider in your house” question, which shows results of “much less likely” and “less likely”. (Data not included for UNSM 2013)

In terms of increasing interest in arachnids, adults/families reported an increased interest in both reading about and observing arachnids (Figure 7). Similarly, youth reported a strong interest in learning more about spiders (Figure 7). Among the youth, we found no differences between boys and girls in their interest to learn more (chi-square = 0.037, df = 1, *p* = 0.85). We did not find a majority of adults/families expressing a change in the likelihood that they would kill a spider (Figure 7).

**Figure 8:**
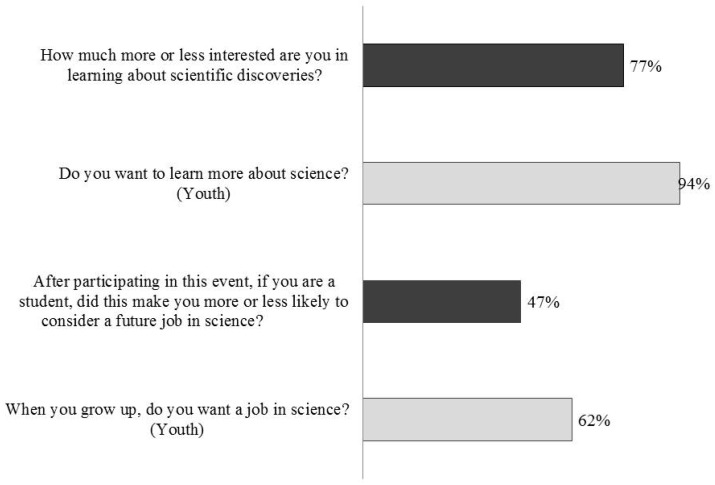
In most cases, the majority of adults and youth reported an increased interest in science and science-related careers as indicated by adult/family (dark bars) responses and youth responses (light bars) to relevant questions. The adult/family questions (top and third down) show the combined adult responses from “much more likely” and “more likely”. The two youth questions (2nd down and bottom) show the proportion of youth responding “yes”. Data included are from BP, UNSM 2014, DMNS, and TZ.

In terms of interest in science, the majority of adults/families and youth responded that they had an increased interest in learning more about scientific discoveries and in learning more about science, respectively (Figure 8). The majority of youth also answered “yes” to wanting a job in science when they grow up (Figure 8). Again, there were no differences between boys and girls in their responses (chi-square = 0.000, df = 1, *p* = 0.98). In contrast to the results from youth, participating in the event did not have a strong impact on adult/family interest in a future job in science (Figure 8).

Survey data regarding science interest from UNSM 2013 could not be combined with the previous datasets, due to substantially different wording. In the UNSM 2013 survey, 98% of respondents agreed to the statement “My family and I learned a lot about science at this event” (36% strongly agree; 62% agree). One hundred percent expressed interest in science based upon responses to the following question “How interested are you (and your family) in learning more about science after this event?” (65% very interested; 31% interested; 4% somewhat interested).

Ninety-four percent of UNSM-surveyed participants reported learning something new. When asked to describe what they learned, responses included: “*Types of silk*”; “*Daddy long legs are not aggressive*”; “*Daddy long legs are not venomous*”; “*Different types of web types of spiders*”; “*Different groups of arachnids*”; “*I have a greatly enhanced appreciation of arachnids*”; “*More about different spiders like reflective eyes and such*”; “*Scorpions can glow*”; “*Pseudoscorpions*”; “*That most spiders don’t see well*”; “*That Thelyphonida is an organism I had not previously heard about*”; “*There are scorpions in Nebraska*”; “*Too many things—but for example, daddy long legs are not spiders*”.

At the BP, the survey question was open-ended: “Did you learn anything new? If so, please briefly describe.” Sample responses include: “*Everything*”; “*All different types of arachnid*”; “*Daddy long legs are not spiders*”; “*Daddy long legs aren’t venomous*”; “*How spiders eat—they drink!*”; “*Most spiders cannot hurt you and are so helpful to our ecosystem. What balance they bring to our world!*”; “*I learn that there are more species of spider..*”; “*I learned a lot about spider nervous system—awesome!*”; “*I learned quite a bit. It was very well done*”; “*Insects + other small creatures are a part of our world and matter too!*”; “*Learned a lot about whip scorpions*”; “*Learned a lot more about arachnids really enjoyed the stations and microscopes*”; “*Not everything is a spider*”; “*Scorpions glow under UV light*”; “*That spiders have different eyes*”; etc. See *Additional Evaluation* section for access to a complete list of responses.

Overall, participants at *Eight-Legged Encounters* recognized the value and importance of free, public, interactive scientific events (“yes” response—76% UNSM; majority written responses from BP included “yes”). When asked why, responses included: “*Absolutely—children need to value and protect the natural world*”; “*Children learn better doing hands-on activities*”; “*Create awareness for science*”; “*Create interest in science*”; “*For them to see first-hand not on a screen*”; “*Kids learn by interacting and discovery*”; “*Absolutely! It should be part of public school curriculum*”; “*Yes! Hands-on activities/events are priceless to the learning process. Not sure I would seek out science-based events without the hands-on learning.*”; “*Yes, because people need to see science in action*”; “*Fun activity, it increased my daughter’s interest in different areas of science*”; “*I believe science is important for everyone to learn*”; “*Yes, to promote science education*”; “*Teaching old and young*”; and “*We have to get kids excited at an early age*”, etc. 

### 3.4. Additional Data and Evaluation

Volunteers overwhelmingly expressed an increased interest in conveying science to the general public and in volunteering at another science-related outreach event. Seventy-four percent of surveyed volunteers (*N* = 47 UNSM 2014; *N* = 40 DMNS) said they were “much more likely” or “more likely” to volunteer at another outreach event. Eighty-four percent responded to being more interested in conveying science to the general public. However, there was little to no impact of volunteering on future career aspirations in either education (20% expressed a higher likelihood of going into an education career) or science (28% expressed a higher likelihood of going into a science career). Overall, volunteers rated their experience highly, with a mean score of 4.5 on a five-point scale, where 1 is “the worst” and 5 is “the best”. Many volunteers felt that interacting with youth was their favorite part of the experience. Open-ended responses about their favorite part of the experience included: “*Watching the kids get excited about learning*”; “*Seeing young kids get really interested in science and something new*”; “*The kids are willing to listen to scientific facts more than I previously thought*”; and “*The excitement on the faces of the kids as they left”*. 

The development of *ELE* was supported by a grant from the National Science Foundation (to E.A.H.), and a complete report of the project, along with more detailed evaluation, can be found on the Center for Advancement of Informal Science Education Website (CAISE [89]). Downloadable materials for activities, photos of the event, yearly evaluation reports, and more background information can additionally be found on the Hebets’ Lab website [90]. 

## 4. Discussion

*Eight-Legged Encounters*—a family-friendly informal science learning event consisting of numerous modular activities developed around the life science core concepts of biodiversity and evolution—was developed, implemented, and tested as an educational model that bridges informal and formal science learning. The Next Generation Science Standards (NGSS) were used as foundations for developing interactive activities that attempted to leverage the public’s perceived fascination with, and emotional connection to, arachnids (whether positive or negative) for science learning. By aligning a scientist’s research and outreach programs (E.A.H.) with formal educational goals (NGSS), we aimed to use regional museums and zoos to engage the public in fun activities that (a) increase the participant’s motivation to learn, (b) contributed to “connected” knowledge across learning contexts, and (c) facilitated the learning of new skills [13]. Though this study primarily reports evaluation data regarding the efficacy of the overall event with respect to engagement and interest, preliminary results nonetheless suggest that our model holds promise for providing a pathway from informal to formal science learning. 

The successful implementation of *Eight-Legged Encounters* (*ELE*) at multiple venues across the country, in combination with results from participant surveys, suggests that arachnids can be effectively leveraged for informal science engagement and learning. Focal *ELE* activities were well attended, and were generally perceived as interesting. The volunteers facilitating the activities, as well as the original artwork scattered throughout the exhibit, were reported to be helpful in engaging audience participation. Across both adults and youth, we found preliminary evidence of increased interest in both arachnids and in science more generally; and a majority of youth reported an increased interest in wanting a job in science. We also found preliminary evidence of participant learning, as demonstrated through answers to open ended survey questions. 

### 4.1. ELE Activities

All of the surveyed *ELE* biodiversity and evolution-focused activity stations attracted participants. The proportion of surveyed participants that visited each station ranged from 59–88% among adults/families, and 40–97% among youth. The slight discrepancy in visitation/participation numbers between adult and youth surveys may be due, in part, to youth respondents not knowing or forgetting the names of specific stations. Personal observations (E.A.H. and P.T.) suggest that youth were almost always with their caregivers. Regardless, there were no activities that appeared unsuccessful in attracting visitors.

The *Path of Predators* consistently received high participation scores. This may not be surprising, as this “activity” actually encompassed eleven distinct stations, increasing the likelihood that visitors encountered at least one. Additionally, *Path of Predator* stations frequently had live animals, making them inherently more attractive (personal observation, E.A.H.). Interestingly, *Microscope Madness* was also highly attended, despite having lower overall interest scores from youth (10% found it “not much or not at all” interesting). Microscopes are a symbol of science and require hands-on engagement. We suspect that the visual presence of microscopes was sufficient to draw in visitors. Upon arrival, however, many participants—youth and adults alike—had a difficult time using the microscope. The difficulties encountered, despite receiving significant help from volunteers, was likely responsible for the relatively lower interest scores among youth. 

In addition to high visitation rates, the majority of all responding adults and youth reported strong interest in all activities, suggesting that they were successful in capturing the visitor’s interest. The *Community Experiment* was one particular activity that we heard about for weeks following the event. This activity was also the one that most closely outlined, and walked participants through, the scientific process and aligns most closely with NGSS Science and Engineering Practices. It was heartening to see that this activity also received high interest scores (>95% of participants found it “very interesting” or “interesting/somewhat interesting”). Unfortunately, this activity also requires a fair amount of (i) prior time, space, and resource commitment—i.e., collecting and housing hundreds of spiders; (ii) materials—i.e., plastic tubs, peat moss, head lamps, granite slabs, stopwatches, forceps, feeder crickets, computer and monitors for data entry, etc.; and (iii) space—i.e., a darkened room to enable “shining for spiders”, as well as multiple experimental stations for conducting the experiment. Nonetheless, this activity, or a modification thereof, works quite well in smaller settings. For example, we have used it with Upward Bound student groups, and we implemented it at the Entomology Department’s annual “Bugfest” event at UNL (evaluation data not included, but can be found in the formal report for this project: see *Results—Additional Data*). Similar activities have also been used with smaller numbers of spiders at summer camps.

Activities that we found to be particularly successful and easy to implement as modules for other events (e.g., science fairs, summer camps, afterschool science clubs, K-12 classroom visits, and hands-on science center activities, among others) included *Create a Chelicerate*, *Assemble and Arachnid*, and *Catch a Moth*. *Create a Chelicerate*, however, can require a large amount of supplies and can get costly, depending upon the size of the anticipated audience. In 2016, for example, the National Science Foundation invited *ELE* to the USA Science and Engineering Festival in Washington, DC, as part of the NSF exhibit. Due to space and monetary limitations, we chose not to bring *Create a Chelicerate*. Instead, we brought the following activities—*Mini Path of Predators* (Amblypygi, Araneae, Scorpiones, and Thelyphonida), *Catch a Moth*, and some wooden puzzles and stuffed animals for toddlers. This combination of activities was ideal for the space and audience, and received high praise from attendees. Unfortunately, due to the crowds (estimated >10,000 people/day over 3 days), the demand on *ELE* volunteers, and the extra required cost, we were unable to conduct evaluation at this event. Nonetheless, this is an example of the modular aspect of *ELE*, as any number of activities can be combined depending on space, volunteers, interest, etc. 

Coincident with our many successes, observations during the initial *ELE* (UNSM 2013) revealed participant frustration with particular activities, such as *Weave a Web—*an activity for which 59% of respondents showed strong interest. In response to both informal observations and formative survey feedback, the activity was modified for the second *ELE* event (see Table 3). Though more successful following its revision, *Weave a Web* was still found to be a challenging activity to successfully implement. Indeed, 61% is the highest interest score this activity ever received. While the revised sewing activity that used different colored yarn and plastic fabric to create an orb web appeared to be effective at conveying the target information—i.e., that spiders use multiple types of silk to weave an orb web—the activity remained difficult for young children. Even for adults, the activity took a lot of time, and was observed to be confusing to many participants. As such, we concluded that the learning outcomes acquired following the completion of the *Weave a Web* activity were not worth the time investment to complete the activity, as this cut into time that could otherwise be used for additional modules. We thus removed *Weave a Web* from subsequent *ELE* events, and replaced it with new silk-related activity stations (i.e., *Build a Burrow* and *Cribellate* vs. *Ecribellate Silk*). 

In summary, evaluation data across five events at four museum and zoo venues suggest that *ELE* activities were successful at both attracting visitors and piquing their interest. Though these activities were developed with NGSS in mind, the task remains to both expand upon these activities by integrating additional NGSS and to better integrate them with formal school activities. Conversations between formal and informal science educations will be crucial moving forward, so that efforts on both sides can be synergistic and complementary. For example, knowing what core concepts are the most challenging to teach could help guide scientists in developing informal learning programs. On the flip side, formal educators can integrate and expand upon informal science programming that uses NGSS as their foundation. We argue that for many of the reasons outlined previously, spiders can make excellent classroom subjects for a range of science studies and can help to weave a web of connectivity between formal and informal science learning.

### 4.2. ELE Volunteers and Artwork

We found that both the volunteers and artwork of *ELE* were effective at engaging visitors. Eighty-five of surveyed participants reported the volunteers to be “very effective” at engaging them with the exhibit, as compared to 68% for artwork. When we combine the responses of “very effective” and “somewhat effective”, however, the scores were 96% and 92% respectively. Though our data do not allow us to disentangle the exact manner in which volunteers or artwork facilitated engagement, we suspect that they worked synergistically and in a sequential fashion. In particular, we propose that the artwork may have drawn in audience members from a distance, as many attendees at the focal venues did not necessarily come for *ELE*, and otherwise may have simply passed by the exhibit. Once audience members were attracted, however, we expect that they were drawn in to the specific activities by volunteers. Future research could test these hypotheses. Specifically, focal observations at future *ELE* events could reveal whether or not participants were more likely to continue their participation in additional *ELE* activity stations following positive interactions. 

Our integration of original, non-scientific artwork, into *ELE* events, created a setting reminiscent of both an art museum and a science festival. While our minimal survey questions certainly suggested that the artwork had an impact on attracting participants, we are particularly interested in future work exploring this aspect of *ELE* further. For example, did the fact that it was original artwork created by an artist with no prior arachnid knowledge matter? Are there distinct impacts of scientific illustration versus more freestyle, interpretive art? Did the artwork draw in visitors that would otherwise have not engaged in the exhibit? While we recognize that the incorporation of art into science is not new, we nonetheless see numerous potential avenues for future exploration of this synergistic approach to science learning.

### 4.3. ELE Families and Youth Impact—Spiders and Science

More than two-thirds of surveyed participants, both adults/families and youth, reported an increased interest in learning more about spiders, reading about arachnids, and taking the time to observe a spider or arachnid. Less than 50% of adults/families surveyed (40%), however, reported being “less likely” or “much less likely” to kill a spider in their house. Interestingly, this response varied significantly across sites (chi-square = 105.8, df = 12, *p* < 0.001). More than 50% of participants at UNSM 2014 (55%) and the BP (58%) reported being “less likely” or “much less likely” to kill a spider as compared to only 34% of participants at both the DMNS and the TZ. Unfortunately, due to the wording of our survey question, the results are difficult to interpret, as we do not know the starting point for any participant. It is possible, and even likely, that a number of participants already would not kill a spider in their home. In such instances, participants would likely choose “the same”—a response that received high scores across all sites (UNSM 2014, 36%; BP, 40%; DMNS, 38%; Toledo Zoo, 50%). Interestingly, the two sites that received the most positive scores for this question (UNSM 2014 and BP) were also sites that either specifically advertised the event (UNSM) and/or had an arthropod focus (BP). As such, it is likely that attendees came specifically to learn about either arachnids (i.e., *ELE* at UNSM) or insects generally (BP). By contrast, *ELE* at the DMNS and the Toledo Zoo was an extra event that most attendees did not know about prior to entering the museum or zoo. As such, the audience at these two events (DMNS and TZ) was likely more diverse in terms of innate interest in arthropods. In the future, it would be interesting to obtain additional information on both the reason for the visit to the venue, as well as on prior behavior towards arachnids in one’s home.

By providing organism-based informal science programming with opportunities for personal experience and with content that resolves existing misinformation and myths, we had aimed to reduce the prejudice-based fear and culturally evolved revulsion often associated with spiders [31]. Indeed, attitudes regarding unpopular animals have previously been shown to increase with increasing knowledge (e.g., bees [91], sharks [92]). Though our preliminary evaluation data are intriguing, we acknowledge that additional studies are required to truly assess the impact of *ELE* on attitudes towards arachnids. Spider-specific survey questions already exist (e.g., [64]) and future work should incorporate and build on this existing knowledge base. Additionally, in order to explore the impact of *ELE* on a more diverse audience, it would be interesting to expand *ELE* events to venues beyond museums and zoos, as these venues are likely to attract visitors with a predisposition towards positive attitudes and interest in arachnids.

While our evaluation cannot address science learning directly, 94% of surveyed participants reported learning something new after having attended *ELE*. Responses to the open-ended questions asking them to describe what they learned primarily included facts about arachnids, such as “*Scorpions can glow*”, “*Daddy long legs are not venomous*”, “*Insects + other small creatures are a part of our world and matter too!*”, and “*That most spiders don’t see well*”. Multiple participants noted learning facts about “daddy long legs” (Order Opiliones) such as that they are not spiders, they are not venomous, and they are not aggressive. Additionally, multiple participants reported learning that scorpions fluoresce, or recited facts regarding spider silk. Aside from these topics, participants mostly reported learning about animals that had previously been unfamiliar to them—e.g., pseudoscorpions, thelyphonids, etc. The number and range of responses to these open-ended questions indicate that participants did indeed increase their knowledge about biodiversity, but a demonstrated increase in knowledge about evolution was not obvious.

Adults and youth alike reported strong increases in their interest in learning about scientific discoveries (77% of adults) and about science generally (94% of youth). Among youth, but not adults, we found a majority of respondents expressing an interest in a future job in science (62%). It is not necessarily surprising that slightly less than 50% of adult/family participants reported being “much more likely” or “more likely” to consider a future job in science, as the career trajectories of those responding were likely already set. Though these results are promising, they are based on only a few questions and are quite broad. Future studies should explore the potential impact of *ELE* on increasing positive science attitudes in more detail.

Across our youth datasets, we found no differences between the responses of boys and girls. Indeed, having implemented arachnid-based informal science activities across multiple venues for more than 20 years (E.A.H.), we have never observed obvious gender-based differences in response to arachnids. While girls may initially shy away and verbally express distress, they are simultaneously often the ones at the front of the line asking to hold the tarantula. Future work should quantify these anecdotal observations to further explore perceived gender differences with regards to attitudes towards arachnids.

### 4.4. ELE Volunteer Impact

Volunteers reported increased interest in communicating science (84%) and in participating in future outreach events (74%). By contrast, however, the majority of volunteers did not express more interest in going into an education or science career (20% and 28%, respectively). It is important to note that volunteers were only surveyed at two of the four events—UNSM and DMNS. At the UNSM 2014, a majority of volunteers were graduate and undergraduate students already studying science. As such, these volunteers were likely to answer “the same” to a question about being more or less likely to go into a particular career. At the DMNS exhibit, volunteers were split between science graduate and undergraduate students, and retired volunteers of the museum. Again, both groups are unlikely to alter their career paths as one group is already committed to a particular career path and the other has already completed their career path.

## 5. Conclusions

This study adds to the few existing peer reviewed research studies that assess arachnid educational interventions [42,43]. Based upon our initial evaluation of *ELE*, it appears as though *ELE* activities can successfully capture the attention and interest of visitors attending Midwestern museums and zoos. In our view, capturing visitor’s interest, especially in a family context, is the first step towards subsequent science learning and engagement. The smallest seed of science interest can provide the sustenance for future science learning. Through the development of fun, hands-on science activities, our aim was to plant that seed. 

While our activities were developed with NGSS in mind, we have not yet conducted any assessment on the impact of these activities on knowledge gain or conceptual understanding relevant to the target standards. It is imperative that future work assesses both potential gains in conceptual understanding, as well as the capacity for students to transfer any putative increased understanding across learning contexts. This study has laid the groundwork, demonstrating that activities can attract, engage, and interest youth and adults in informal settings. Now it is time to iteratively evaluate the impact of *ELE* on science understanding, and to revise activities accordingly. Though *ELE* was developed as a model to bridge formal and informal learning environments, much work remains to be done to test and validate this model. 

We hope that *Eight-Legged Encounters* has contributed, and will continue to contribute, to the STEM learning ecosystems in regions throughout the Midwest. In Nebraska, we know that informal science learning opportunities vary greatly by place, and this is likely true throughout the region. In more urban areas, while there may be zoos and museums present, access is sometimes a barrier for youth who lack financial resources. As mentioned earlier, this makes museum and zoo venues potential barriers to inclusive informal science learning. In urban areas in Nebraska, 21st Century Community Learning Centers (CLCs) have filled that gap for some youth in low income Title I schools [93]. Programs run through the CLCs bring community and university partners together in afterschool learning on a broad range of subjects, including science. Indeed, this is an additional way in which *ELE* is contributing to the Lincoln, NE STEM learning ecosystem, as *ELE* activities have been used in such programs. Unfortunately, there are fewer informal science learning opportunities in rural areas [94], but an ecosystem is currently under development via University of Nebraska Cooperative Extension, 4H, EPSCOR, and public libraries who are working to bring more informal science opportunities to western rural Nebraska [95]. To date, *ELE* modules have already been used in afterschool science clubs, in summer science camps, in regional science fairs, and in science days at local science, or children’s museums (NE), and we hope to continue to expand across learning contexts and venues in an effort to use arachnids to bridge science learning experiences for youth (and adults) [96]. 

Finally, we hope that *ELE* can be a model for other scientists, as well as informal science educations: a model of how to leverage organismal biology for informal science learning; a model of how to align formal and informal learning goals; a model of how to make connections and create collaborations among institutions within a community. We also hope that this study can act as a resource for science educators that can be expanded and shared in a way that continues to engage the public and grow interest in arachnology and in science.

## Figures and Tables

**Table 1:** Overview of focal study questions associated with *Eight-Legged Encounters*.

How interested were participants in distinct activities?
How effective were the volunteers and artwork at engaging visitors?
Did participating in the event improve positive attitudes towards spiders?
Did participating in the event increase science interest?

**Table 2:** Arthropod Classification and Taxonomy Activities, alignment with NGSS (Disciplinary Core Ideas, Science & Engineering Practices) and brief descriptions. Superscript abbreviations represent the venues where activities were incorporated (Butterfly Pavilion, CO 2013^BP^; University of Nebraska State Museum—Morrill Hall, NE^UNSM 2013; 2014^; Denver Museum of Nature and Science, CO 2014^DMNS^; Toledo Zoo 2017^TZ^).

Module	NGSS	Background and Activity Description
*What is an Arthropod?* *^(BP; UNSM 2013, 2014; DMNS)^*	LS1.A: Structure & Function LS4.A: Evidence of Common Ancestry & Diversity LS4.D: Biodiversity & Humans Asking Questions and Defining Problems	*Background:* Arthropods are the most diverse group of animals on our planet, and they include the insects, crustaceans, and chelicerates.
		*Activity:* Game. Participants try to place plastic model arthropods in their appropriate square based upon external body features—e.g., six legs vs. eight legs vs. ten legs.
*Assemble an Arachnid* *^(BP; UNSM 2013, 2014 TZ)^*	LS1.A: Structure & Function LS4.D: Biodiversity & Humans	*Background:* Arachnids are surprisingly diverse, with 11 different living orders that vary in size, structure (form), sensory capacity, habitat, and behavior.
		*Activity:* Coloring, cutting, taping/pasting. Participants create their own novel arachnid by choosing a front and back body part to color and put together.
*Create a Chelicerate* *^(BP; UNSM 2013, 2014; DMNS;TZ)^*	LS1.A: Structure & Function LS4.D: Biodiversity & Humans Developing and Using Models	*Background:* Arachnids are arthropods that are closely related to horseshoe crabs and sea scorpions. They all share a general body plan: 2 body parts, 4 pair of walking legs, a pair of chelicerae (mouthparts), and a pair of pedipalps.
		*Activity:* Clay creation. Participants build their own chelicerate using air dried clay and pipe cleaners of various color representing different appendages.

**Table 3:** Spider and Silk Activities, alignment with NGSS (Disciplinary Core Ideas, Science & Engineering Practices, Cross-Cutting Concepts), and brief descriptions. Superscript abbreviations represent the venues where activities were incorporated (Butterfly Pavilion, CO 2013^BP^; University of Nebraska State Museum—Morrill Hall, NE^UNSM 2013, 2014^; Denver Museum of Nature and Science, CO 2014^DMNS^; Toledo Zoo 2017^TZ^).

Module	NGSS	Rationale and Activity Description
*Build a Burrow* *^(UNSM 2014)^*	LS1.A: Structure & Function LS1.D: Information Processing LS4.C: Adaptation LS4.B: Natural Selection Developing and Using a Model	*Background:* The first spiders did not build orb webs to catch prey out of the air, but instead built silk-lined burrows, often with silken trip lines radiating away from the burrow’s lip. Spiders can detect vibrations through these silken trap lines. *Activity:* Craft & Game. Participants build a silk-lined burrow using fishing line as silk, and foam door hangers and snap bracelets as burrows. Once built, a prey encounter is simulated: electric toothbrushes are used to stimulate one of the trip lines, and the participants are challenged with detecting which trip line was triggered.
*Catch a Moth* *^( BP;UNSM 2013, 2014; DMNS;TZ)^*	LS1.A: Structure & Function LS1.D: Information Processing LS3.B: Variation of Traits LS4.B: Natural Selection LS4.C: Adaptation	*Background:* The “bolas spiders” provide an unusual example of adaptation with respect to forging. Adult females produce a chemical that mimics the pheromones of local moths. As male moths approach what they expect to be a female, the hunting spider swings a line of silk with a sticky droplet at the end to capture the moth out of the air [86]. *Activity:* Game. Participants swing a “bolas”—a string with a ping pong ball covered in Velcro—at hanging moths covered in matching Velcro patches to try to catch them out of the air. We use felt moths modeled after two species that are target prey.
*Sticky vs. Wooly Silk* *^(UNSM 2014)^*	LS1.A: Structure & Function LS3.B: Variation of Traits LS4.B: Natural Selection LS4.C: Adaptation ETS1.C: Optimizing the Design Solution Developing and Using a Model Asking Questions & Defining Problems	*Background:* Spiders have evolved two primary ways to make their webs sticky. Cribellate spiders comb out their silk using specialized structures to give it adhesive properties that rely on Van der Waals forces, while Ecribellate spiders add sticky glue droplets to their silk. *Activity:* Experiment. Participants are provided two pieces of yarn. With one piece, they add droplets of glue along the length and then drop confetti (i.e., prey) on the yarn. With the other piece, they use an eyebrow brush to comb out the yarn and similarly drop confetti on top. They then lift up the two pieces of yarn and observe their respective abilities to capture prey.
*Tissue Paper Flower* *^( BP; UNSM 2013, 2014; DMNS)^*	LS4.B: Natural Selection LS4.C: Adaptation Developing and Using a Model	*Background:* Some crab spiders can change color to match the flower on which they sit. They ambush predators, waiting to grab insects that visit the flower. *Activity:* Craft. Using different colored crate paper and pipe cleaners, participants create their own flower. Once created, they pick a crab spider—either white or yellow—and place it on the flower. Volunteers discuss with them the importance of camouflage for these ambush predators.
*Weave a Web* *^(BP; UNSM 2013, 2014)^*	LS1.A: Structure & Function LS3.B: Variation of Traits Structure & Function	*Background:* Orb webs—i.e., “classic” spider webs—are complex structures that are created using different silk types with different properties (e.g., strength, toughness, elasticity), generated from different silk glands. *Activity:* Sewing. Participants use yarn of different color, plastic canvas, and children’s sewing needles to sew a spider web pattern. Different colored yarn is used to signify the different types of silk.
*Sound Station* *^(BP; UNSM 2013)^*	LS1.A: Structure & Function LS1.D: Information Processing LS4.D: Biodiversity and Humans	*Background:* Many spiders “sing” by creating vibrations that can be sent through various substrates—e.g., leaves, grass, pine needles. *Activity:* Listen. Participants are invited to put on headphones and listen to the sounds generated by spiders. Laminated photographs of the structures used to generate these sounds accompany this station.
*Read Aloud* *^(BP; UNSM 2013, 2014)^*	*Not Applicable*	*Background:* Toddlers need an opportunity to let out energy, play, and explore. This can be accomplished in a manner that simultaneously exposes them to science-related content. *Activity:* Listening and play. Toddlers are invited to play with arthropod stuffed animals, puppets, and puzzles. A variety of arachnid-themed children’s books are available to look through, and volunteers read books aloud at intervals throughout the event.

**Table 4:** *Path of Predators* Activities. All eleven stations were implemented at four of the five focal events (Morrill Hall, NE 2013 and 2014; Butterfly Pavilion; and Denver Museum of Nature and Science). At the Toledo Zoo the *Mini Path of Predators* was implemented (Amblypygi, Araneae, Scorpiones, Thelyphonida). Disciplinary Core Ideas (NGSS) covered in the *Path or Predators* activities include: LS1.A: Structure and Function; LS1.B: Growth and Development, LS1.D: Information Processing; LS2.A: Interdependent Relationships in Ecosystems; LS4.B: Natural Selection; and LS4.C: Adaptation; LS4.D: Biodiversity and Humans. Cross-Cutting Concepts include Scale, Proportion, and Quantity and Science Addresses Questions about the Natural and Material World.

Order	Common Names	Activity Description
Acari	Ticks and Mites	*Ectoparasites*: Use the digital handheld microscope to look closely at these ectoparasites—animals that feed on other animals from the outside. Can you tell which individuals had a recent blood meal? Have you ever had one feed on you?
Amblypygi	Whip spider; Tailless whip scorpion	*Ambly Reach*: Use your sense of touch to explore a new environment—reach into a box and guess what is inside. Rainforest Café*: Experience what it would be like to get all of the information about your surroundings from an appendage—reach into openings in this artificial tree trunk and compare what you feel with the “menu” provided. Ambly Wave*: Learn how amblypygids fight one another and how scientists discovered that they use air particle displacement to determine who wins the fight. Try your hand at an amblypygid battle by vibrating your “antenniform leg” as many times as possible over an opponent’s “sensory hair”.
Araneae	Spider	*Eat like an Arachnid ^1^*: Arachnids digest their food externally prior to ingestion, and then use their sucking stomach to pull in the liquefied food. Eat like an arachnid by sucking up applesauce through a straw. Spider Senses*: Learn about spider’s senses, in particular, their ability to detect vibrations in webs. How good would you be at detecting prey? While blindfolded, place your fingers on the different web strings and determine which one is being vibrated.
Opiliones	Harvestmen; Daddy-long-legs (in USA)	*Which one of these is not like the other?* The animals called “daddy long legs” in the United States are actually not spiders, do not have venom, and cannot bite humans. One easy way to distinguish spiders from harvestmen (the preferred common name) is that harvestmen look like they only have one body part (though they really have two). Look at the images on the poster and see how many harvestmen you can find.
Palpigradi	Micro whipscorpion	*Blind Maze*: Palpigrades are tiny blind arachnids that navigate their world using senses other than vision. Test how good you might be at navigating the world using only touch—put on a blindfold and use your fingers to try to complete a three-dimensional maze.
Pseudo-scorpiones	False scorpion; Book scorpion	*Phoresy*: Pseudoscorpions are notorious for hitching rides with other animals—a behavior called phoresy. Experience what it would be like to hitch a ride by sitting on a skate board and using long metal pincers to grab on to a friend as they pull you along.
Ricinulei	Hooded tick spider	*Egg & Spoon Relay*: Male ricinulids transfer sperm to females using a specialized basket-like structure on their third walking legs. They transfer their sperm to a location on the female that sits in between the two body parts. Ricinulids also lack eyes, and are thus blind. Try your hand at ricinulid sperm transfer by transferring sperm (i.e., actually an egg) to a female ricinulid (constructed out of foam balls and empty toilet paper rolls) using a spoon; now try it blindfolded.
Schizomida	Short tailed whip scorpion	*Digging for Prey*: Schizomids are small predatory arachnids often found in leaf litter. See what prey are available to schizomids by digging through one of the tubs of leaf litter. Forceps, vials, and hand lenses are available for those interested in catching and observing their potential prey.
Scorpiones	Scorpion	*Scorpion Shine*: All scorpions have beta-carboline and 4-methyl, 7-hydroxycoumarin in their cuticle, which causes them to fluoresce under ultraviolet light. Shine a blacklight flashlight on a scorpion. Why do you think they fluoresce? Stingers and Pincers*: There is often a negative relationship between the size of a scorpion’s pincers and the size of its venom bulb. Scorpions that rely more on their pincers for prey capture tend to rely less on their venom. Test how effective pincers (i.e., forceps) of varying size are at capturing prey of varying size (i.e., seeds).
Solifugae	Wind scorpion; Sun spider; Camel spider	*Eat like an Arachnid*: Arachnids digest their food externally prior to ingestion and use their sucking stomach to pull in the liquefied food. Eat like an arachnid by sucking up applesauce through a straw.
Thelyphonida	Whip Scorpion; Vinegaroon	*Chemical Defenses*: Vinegaroons get this common name from their defensive behavior—spraying liquid from glands at their hind end towards predators. The liquid is made up primarily of acetic acid, and thus smells like vinegar. Take a cotton ball, squirt liquid on it from the bottle provided. Hold the cotton ball up to your nose to see how well this strategy would work if you were the predator.

* Activities new to the Toledo Zoo exhibit (2017); ^1^ This activity has been used for the Araneae station (Toledo Zoo) as well as the Solifugae station (NE & CO 2013–2014).

**Table 5:** Hands-on Science Activities’ alignment with NGSS (Disciplinary Core Ideas, Science & Engineering Practices, Cross-Cutting Concepts), and brief descriptions.

Module	NGSS	Rationale and Activity Description
*Microscope Madness*	LS1.A: Structure & Function LS1.B: Growth and Development LS3.B: Variation of Traits LS4.D: Biodiversity & Humans Asking Questions & Defining Problems Structure & Function	*Background:* Manipulating organisms under a microscope, making observations, and comparing what you see to scientific material is common practice for many scientists. *Activity:* Participants have an opportunity to be a scientist by taking specimens out of vials, placing them under a microscope, and looking for particular structures associated with a “key” that is provided.
*Community* * *Experiment*	LS1.A: Structure & Function LS2.A: Interdependent Relationships in Ecosystems LS4.B: Natural Selection LS4.C: Adaptation Asking Questions & Defining Problems Planning & Carrying out Investigations Analyzing & Interpreting Data Engaging in Arguments from Evidence	*Background:* The scientific process involves making an observation, generating a hypothesis and associated predictions based upon that observation, and designing and carrying out an experiment to test the hypothesis. The experiment itself includes data collection, entry, analysis, and ultimately interpretation. *Activity:* Participants become scientists as they participate in an experiment aimed at testing a hypothesis about wolf spider foraging behavior (see Sample Activity for details). Specifically, participants collect their own spider and conduct a behavioral foraging assay in one of two pre-determined treatments. They enter their data in a community spreadsheet and observe the current results on a graph that updates in real time.

* Given space and volunteer constraints, this module was only conducted at Morrill Hall (UNSM, 2013, 2014).

**Table 6:** Overview of venues of *ELE* and sample sizes for evaluation data. Hosting sites included the University of Nebraska State Museum’s Morrill Hall (UNSM 2013 & 2014) in Lincoln, NE; the Butterfly Pavilion (BP) in Westminster, CO; the Denver Museum of Nature and Science (DMNS) in Denver, CO; and the Toledo Zoo (TZ) in Toledo, OH. Youth surveys were not collected at UNSM 2013, BP, or TZ (see *NA—Not Applicable*).

	UNSM 2013	UNSM 2014	BP	DMNS 2014	TZ 2017	TOTAL
Adult/Family Participant Survey	100 ^a^	53 ^b^	50 ^c^	209 ^d^	38 ^e^	350
Youth Participant Survey	*NA*	37 ^f^	*NA*	128 ^g^	*NA*	165

^a–e^ Different surveys were used across all five events (see Supplementary Materials). ^f,g^ Different youth survey were used at UNSM 2014 and DMNS.

## Supplementary Materials

The following are available online at http://www.mdpi.com/2075-4450/9/1/27/s1, Figure S1: *Path of Predators* stamps; Figure S2: *Path of Predators* clay sculptures; *Path of Predators* Activity Booklet; *Mini Path of Predators* Activity Booklet; Survey a; Survey b; Survey c; Survey d; Survey e; Survey f; Survey g; and Volunteer Survey.
