# Supplementary material for: Eight-Legged Encounters—Arachnids, Volunteers, and Art help to Bridge the Gap between Informal and Formal Science Learning
Source: Insects. 2018 Feb 26;9(1):27. doi: 10.3390/insects9010027 (PMC5872292; doi:10.3390/insects9010027)
Supplement: Supplementary file 1 [file insects-09-00027-s001.zip › Eileen Hebets/Survey g.pdf]

Eight-Legged Encounters Child Survey
(Denver Museum of Science and Nature)

We would like to learn more about your experiences at this event today. Please take some time to let us know your thoughts.

How much did you like the…	A lot	A little	Not much or not at all	Not Interesting
**1.** Create a chelicerate	○	○	○	○
**2.** Tissue paper flower	○	○	○	○
**3.** Assemble an arachnid	○	○	○	○
**4.** Path of predators booklet	○	○	○	○

5. Do you want to learn more about spiders?

○ Yes

○ No

6. Do you want to learn more about science?

○ Yes

○ No

7. When you grow up, do you want a job in science?

○ Yes

○ No

8. How old are you?

9. (INTERVIEWER MAKE NOTE OF THIS) What is your gender?

○ Male

○ Female

Any other comments
