# Supplementary material for: Eight-Legged Encounters—Arachnids, Volunteers, and Art help to Bridge the Gap between Informal and Formal Science Learning
Source: Insects. 2018 Feb 26;9(1):27. doi: 10.3390/insects9010027 (PMC5872292; doi:10.3390/insects9010027)
Supplement: Supplementary file 1 [file insects-09-00027-s001.zip › Eileen Hebets/Mini Path of Predators.pdf]

Did you know... that there are eleven major groups of living arachnids? (i.e. eleven Orders in the Class Arachnida)!

Discover the wonderful world of arachnids with us!

Find four arachnid stations. Get a STAMP in every space after you visit each of the four stations and complete the associated activities.

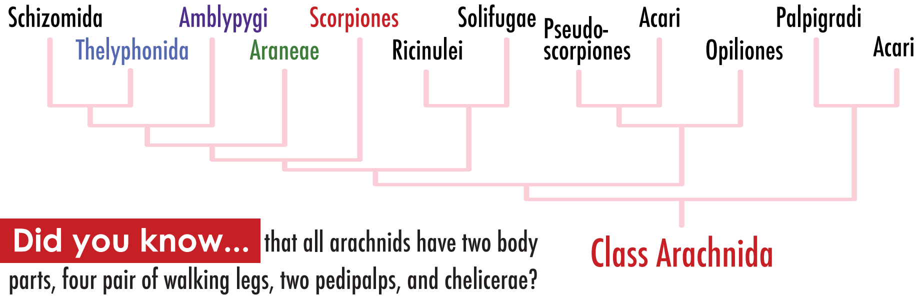

Did you know... that all arachnids have two body parts, four pair of walking legs, two pedipalps, and chelicerae? (don't know what those words mean? LOOK THEM UP!)

ORDER ARANEAE (SPIDERS)

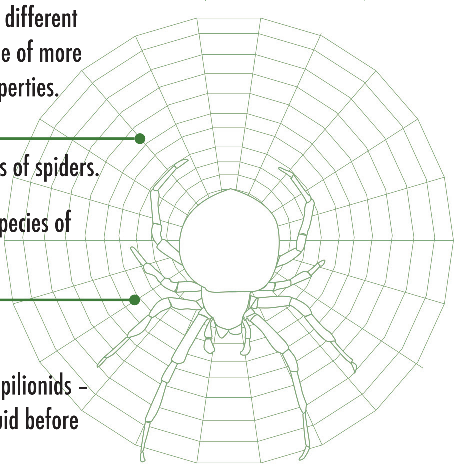

NOTICE all of the different ways that spiders use silk.

DID YOU KNOW... that spiders have more than seven different types of silk glands? A single spider web can be made of more than four types of silk or glue, each with distinct properties.

LOOK at the different shapes, sizes, and colors of spiders.

DID YOU KNOW... that there are more than 45,000 species of spider in the world?

EAT like a spider!

DID YOU KNOW... that nearly all arachnids (except Opilionids – common name harvestmen) turn their prey into liquid before sucking it up in their mouths?

AT HOME – Take a walk outside and find three spider webs that have different shapes. Why do you think the webs are shaped differently? Can you find the spiders who live in the webs? Do the spiders differ in size, shape or color? How?

ORDER AMBLYPYGI

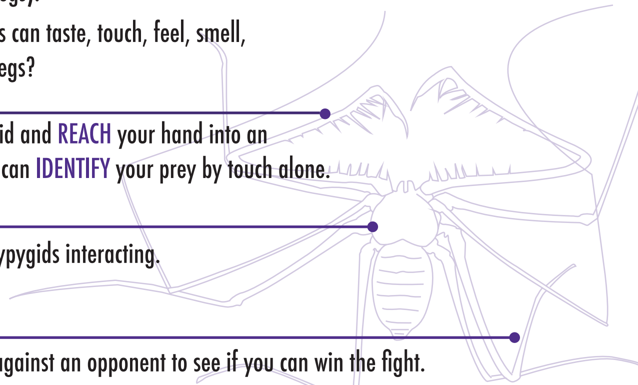

LOOK at the incredibly long 1st pair of legs on these arachnids (called antenniform legs).

DID YOU KNOW... that amblypygids can taste, touch, feel, smell, and listen with their antenniform legs?

PRETEND you are an amblypygid and REACH your hand into an opening to see if you can IDENTIFY your prey by touch alone.

OBSERVE the behavior of amblypygids interacting.

GRAB a "leg" and face off against an opponent to see if you can win the fight.

Did you know... that amblypygids use long, thin hairs on their walking legs (called trichobothria) to detect the movement of air particles made by their opponent's leg waving?

AT HOME– Grab a notebook and pencil and find a cozy spot outdoors. Sit down and close your eyes. Use your senses other than vision to explore the world around you. Describe what you hear, smell, taste, and touch – write it down. Do this at three different times of day. How does it change?

ORDER THELYPHONIDA (VINEGAROON)

TAKE a squirt bottle from the table and **GRAB** a cotton ball.

SQUEEZE the liquid from the bottle onto the cotton ball.

SMELL the cotton ball. Do you recognize the smell? What does it remind you of?

IMAGINE that you are a predator about to eat one of these creatures. A stream of vinegar-like liquid is shot at you from its hind end. Would you continue to pursue this prey?

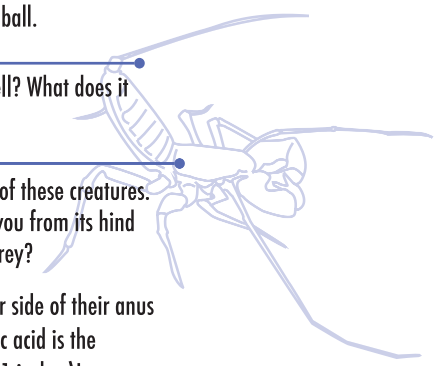

DID YOU KNOW... that vinegaroons have glands on either side of their anus (their back end) that produce defensive chemicals? Acetic acid is the main component and they can spray as far as 80cm (>31 inches)!

AT HOME – Go outside and try to catch five arthropods (insects, arachnids, crustaceans, or centipedes and millipedes). Do any of them have an obvious defense? Are they camouflaged? Are they brightly colored, potentially warning predators off? Do they make a sound or produce a smell when startled?

ORDER SCORPIONES

TURN ON a UV light (black light) and observe how scorpions fluoresce.

DID YOU KNOW... that the cuticle of all scorpions fluoresces and scientists still are not sure why? Maybe YOU can be the scientist to figure out this evolutionary mystery!

NOTICE that some scorpions have BIG pedipalps and small bulbs at the end of their tail while others have small pedipalps and BIG bulbs at the end of their tails. The bulbs contain venom.

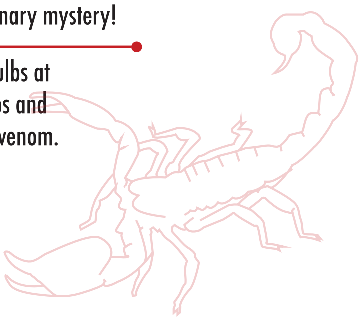

PREDICT which scorpion is more dangerous.

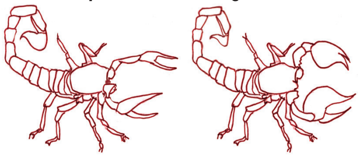

DID YOU KNOW... that scorpions don't always use the venom in their tail to capture prey?

PUT ON SOME PINCERS (SCORPION PEDIPALPS) AND TRY TO CAPTURE THE PREY!

AT HOME- Search on the computer for research articles or websites about scorpion venom. Components of scorpion venom are being studied as potential treatments for a variety of human diseases and disorders. Can you find out which ones? What are some of the most promising treatments?

UNIVERSITY OF Nebraska Lincoln

NSF

Mini PATH OF PREDATORS

A JOURNEY THROUGH THE LIVING ARACHNIDS

by Dr. Eileen A. Hebets, School of Biological Sciences, University of Nebraska-Lincoln

Illustrations by Pawl Tisdale

Funded in part by the National Science Foundation; the College of Arts and Sciences, UNL; and the Office of Research and Economic Development, UNL
