# Supplementary material for: Eight-Legged Encounters—Arachnids, Volunteers, and Art help to Bridge the Gap between Informal and Formal Science Learning
Source: Insects. 2018 Feb 26;9(1):27. doi: 10.3390/insects9010027 (PMC5872292; doi:10.3390/insects9010027)
Supplement: Supplementary file 1 [file insects-09-00027-s001.zip › Eileen Hebets/Path of Predators Activity Booklet.pdf]

Grab a pencil — **GO** on a journey through the exhibits — **Discover** the wonderful world of arachnids

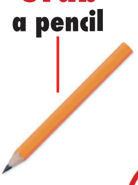

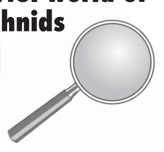

Find all eleven stations. Place a stamp in the circle of the arachnid order as you complete the booklet activities.

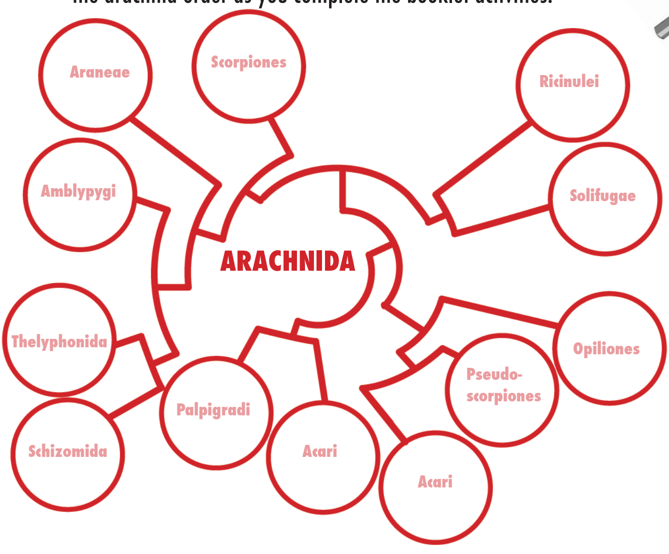

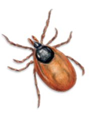

ORDER ARANEAE (SPIDERS)

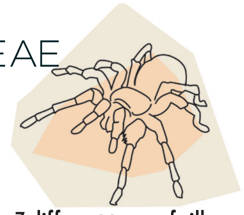

Look at the different colors, shapes, sizes, and lifestyles of spiders.

Imagine what kind of spider you would be.

Draw it below.

Notice that spiders have up to 7 different types of silk glands, each of which produces silk with different properties.

Draw a spider's web between these branches. What types of silk would be used? **Circle** the names of the glands that produce them.

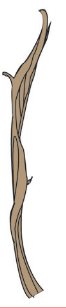

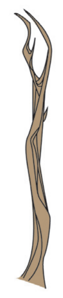

Major ampullate gland
Minor ampullate gland
Flagelliform gland
Aciniform gland
Aggregate gland
Tubuliform gland
Pyriform gland

At Home – Take a walk outside, or even around your house and find three spider webs that differ in their shape. Can you find the spiders who live in the webs? Do the spiders differ in size, shape or color?

ORDER AMBLYPYGI

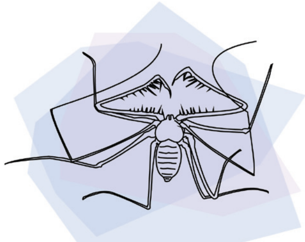

Look at how long the 1st pair of legs are compared to the other walking legs. These animals taste, smell, touch, and listen with their legs.
Reach into one of the boxes on the table and **Feel** the object(s) inside.
Write three words that best describe what you feel.

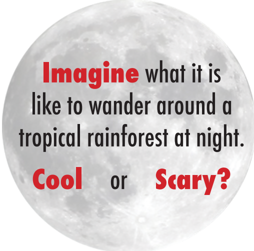

Imagine what it is like to wander around a tropical rainforest at night.
Cool or **Scary?**

All amblypygids are nocturnal and leave their home refuges at dusk to forage. They return to their refuge at dawn. Many are known to travel far distance and some scientists believe that they use their sense of smell to find their way around. **How** might you test this idea?

At home – Grab a notebook and pencil and find a cozy spot outdoors. Sit down and close your eyes. Use your senses other than vision to explore the world around you. Describe what you hear, smell, taste, and touch – write it down. Do this at three different times of day.

ORDER THELYPHONIDA (VINEGAROON)

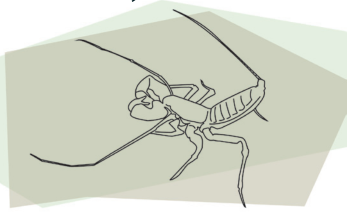

Take a squirt bottle from the table and **Grab** a cotton ball.
Squeeze the liquid from the bottle onto the cotton ball.
Smell the cotton ball. Do you recognize the smell?
What does this remind you of?

Imagine that you are a predator about to eat one of these creatures when a stream of vinegar-like liquid is shot at you from its hind end. Would you continue to pursue this prey? **YES** or **NO**

Vinegaroons have glands on either side of their anus (hind end) that produce defensive chemicals. Acetic acid is the dominant component. They can spray as far as 80cm (>31 inches). Can you think of any other animals that have a protective chemical defense? **Sketch** the animal in this space.

At home – Go in your backyard or to a park and catch five arthropods. Do any of them have an obvious defense? Are they camouflaged? Are they brightly colored, potentially warning predators off? Do they make a sound when startled? Did you notice any foul smells upon collecting them? Make notes on your observations and start a list of arthropod defenses. (Be sure to release your animals so others can learn from them as well!)

ORDER SCHIZOMIDA

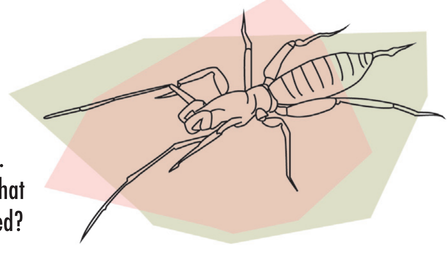

Notice the similarity between these arachnids and the thelyphonids and amblypygids. Can you tell how they differ from the other arachnids you have seen today?

Schizomids are small arachnids about which we know very little. They are found in the leaf litter and frequently go unnoticed. What other small leaf litter animals might also frequently go unnoticed?

Pick up a pair of soft forceps from the table.
Find an available tub of leaf litter.
Dig through the litter. Do you see any animals?
Write down the first two animals you find.

If you did not find any animals, write down the names of animals you think you might find digging through leaf litter. **Which** of these do you think schizomids might eat?

At home – Grab a garbage bag and go out to the woods. Pick up a few armfuls of leaf litter and put them in the bag (check yourself for ticks afterwards). Bring the bag home and place a few handfuls of your collected litter into a colander. Put the colander in a plastic tub. Shake the colander and then place it directly under a light. Monitor the tub underneath to **SEE** what critters climbed or fell into the tub.

ORDER SCORPIONES

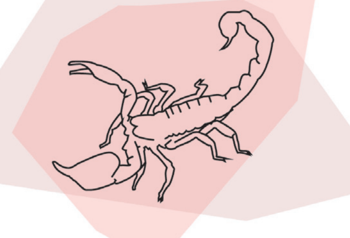

Notice that some scorpions have BIG palps and small bulbs at the end of their tail while others have small palps and BIG bulbs at the end of their tails.
What do you think is kept in those bulbs?
Which scorpion do you think is most dangerous? (big palps/small bulb or small palps/big bulb.)
Observe a scorpion under a black light. What do you notice?
All scorpions fluoresce, but scientists do not know how or why fluorescence has evolved in scorpions. Can you think of ways that scorpions might benefit from fluorescing?
List them in the space below.
Maybe **YOU** can be the scientist to figure out this evolutionary mystery!

At home – Search on the computer for research articles or websites about scorpion venom. Components of scorpion venom are being studied as potential treatments for a variety of human diseases and disorders. Can you find out which ones?

ORDER SOLIFUGAE

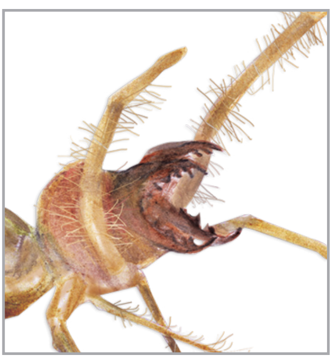

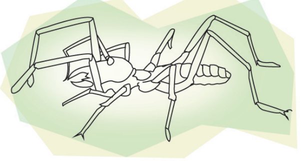

Did you know that solifugids are called "camel spiders" because of the large hump on their 1st body part? This hump houses the huge muscles that operate their jaws.

Look at the huge scissor-like chelicerae on these incredible predators. They use these formidable mouthparts to shred their prey. These animals are sometimes referred to as the "great white sharks" of the arachnid world.
How do you suppose these animals eat their food?

For most arachnids (except Opiliones) digestion is initiated outside of the body. Digestive fluid is regurgitated onto a captured prey item. When the prey is liquefied, it is sucked into the mouth with the help of the sucking stomach.

Take a cup of "food" from the table top. **Grab** a straw as well. Use the straw to **Suck** up the food. How does it **Taste**? Can you tell what kind of food you are eating?

At home – With your parents help and permission, put ½ of your dinner in a blender one night. Taste and compare the solid and liquid versions. Does food taste different when it is liquid versus solid? Is it easier or more difficult to eat in one form or the other?

ORDER PALPIGRADI

Imagine a life without sight. Palpigrades do not have eyes.

Close your eyes and try to draw the tail on the palpigrade below.

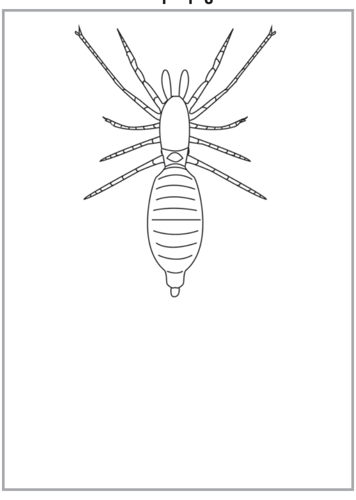

Scientists know very little about these creatures. Isn't it exciting to think about how much there is left to discover?

ORDER RICINULEI

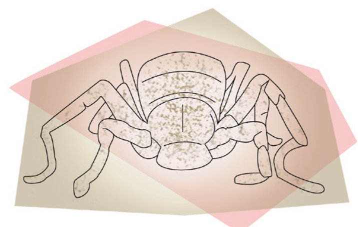

Examine a ricinulid under the microscope. Pay close attention to the 3rd pair of walking legs. Do you **Notice** anything strange?

The 3rd pair of walking legs on males are modified into structures used for mating. Modifications near the tips of the 3rd legs hold and transfer a spermatophore (a packet housing sperm) to a female.

At home – Gather some friends and family together; grab some eggs and spoons and have a good old fashion egg and spoon race. Imagine that you are male ricinulids carrying your spermatophores to your mates.

ORDER PSEUDOSCORPIONES

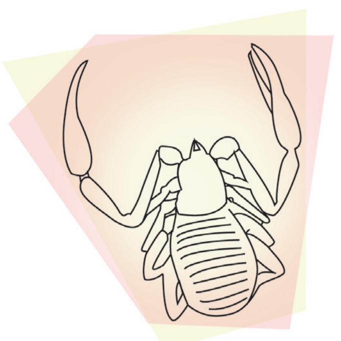

Did you know that these small arachnids can produce silk? They have spinnerets on their chelicerae. They also have venom in their pedipalps!
How do you suppose these small predators use their silk?
Write down three ways you might use silk if you were a pseudoscorpion.

Unlike spiders, pseudoscorpions do not use silk to capture prey. Instead, they use it to produce cocoon-like silken chambers in which they shed their skin (moult), hibernate, and lay and protect their eggs. Some also use silk to **"Hang On"** as they hitch a ride. Pseudoscorpions are notorious for hitchhiking on a variety of organisms. If you could, what animal would you most like to hitch a ride on?

At home – Set up a race course outside and find a stopwatch. Run the course as fast as you can forward. Time yourself and write down the time. Now turn around and run the same course backwards. Compare the times. How good are you at running backwards? Pseudoscorpions can run backwards at surprising speeds

ORDER ACARI (MITES & TICKS)

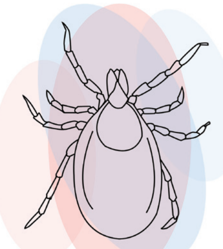

Picture a group of organisms that live in habitats as diverse as: decomposing matter, plants, fresh water, salt water, and inside animals (for example in the nasal passages of seals, the lungs of owls, the digestive system of sea urchins, among others). These are the Acari – they are predators, scavengers, detritivores, and fungivores and are the most diverse of the living arachnid orders!

Many mites and ticks live as ectoparasites on the outside of a host animal. For example, there are tiny parasitic mites that are frequent inhabitants of human eyelashes!

Look at the ectoparasitic ticks on display. Can you tell which ones have had a recent blood meal?

Think about a time that you have encountered a tick. If you have not yet, ask your parents. Where was it? What was it doing? **Describe** the encounter in a few sentences here.

At home – Next time you find a tick on yourself or your dog, before disposing of it, take a close look. Can you determine what species it is? Can you see two body parts? Eight legs?

ORDER OPILIONES (HARVESTMEN)

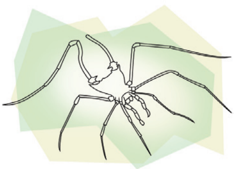

Examine the arachnids below.
Circle all of the opilionids. How many are there?

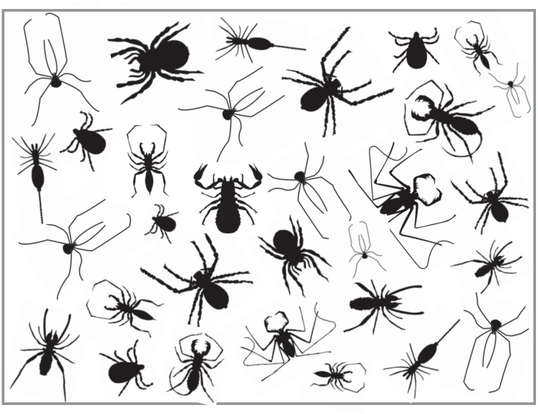

Many people think that opilionids are spiders, but they are not.
List the ways in which opilionids are different from spiders.

At home – Go outside in later summer and collect 5–10 opilionids from the side of your house or on vegetation surrounding your house. Get a large Tupperware container and put all of your collected individuals in together. What happens? Do they attack and eat each other? Most arachnids will readily cannibalize one another, but opilinoids are frequently found together in large aggregations. It is thought that they group together for protection.

PATH OF PREDATORS

A JOURNEY THROUGH THE LIVING ARACHNIDS by EILEEN A. HEBETS

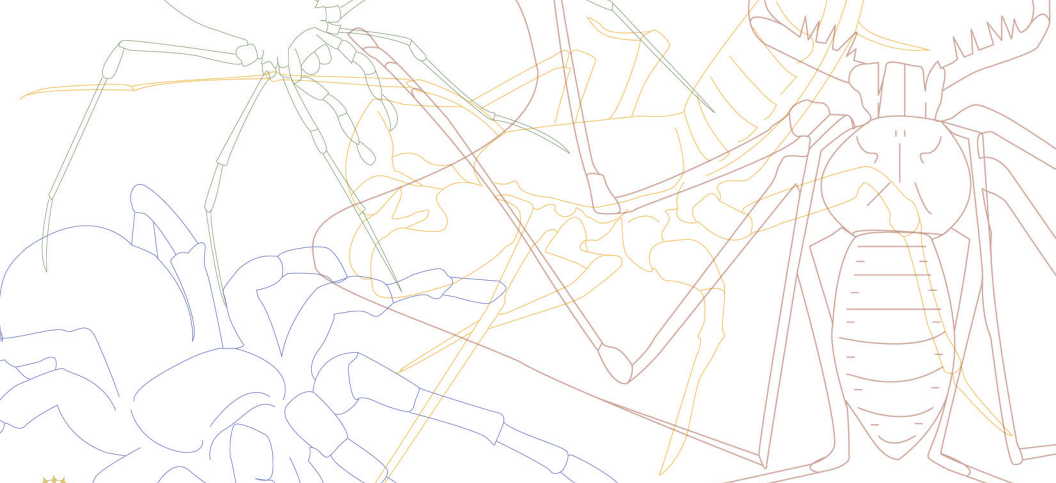

Funded in part by the National Science Foundation

Illustrations by Pawl Tisdale
