# Supplementary material for: Eight-Legged Encounters—Arachnids, Volunteers, and Art help to Bridge the Gap between Informal and Formal Science Learning
Source: Insects. 2018 Feb 26;9(1):27. doi: 10.3390/insects9010027 (PMC5872292; doi:10.3390/insects9010027)
Supplement: Supplementary file 1 [file insects-09-00027-s001.zip › Eileen Hebets/Supplemental Figure 1.pdf]

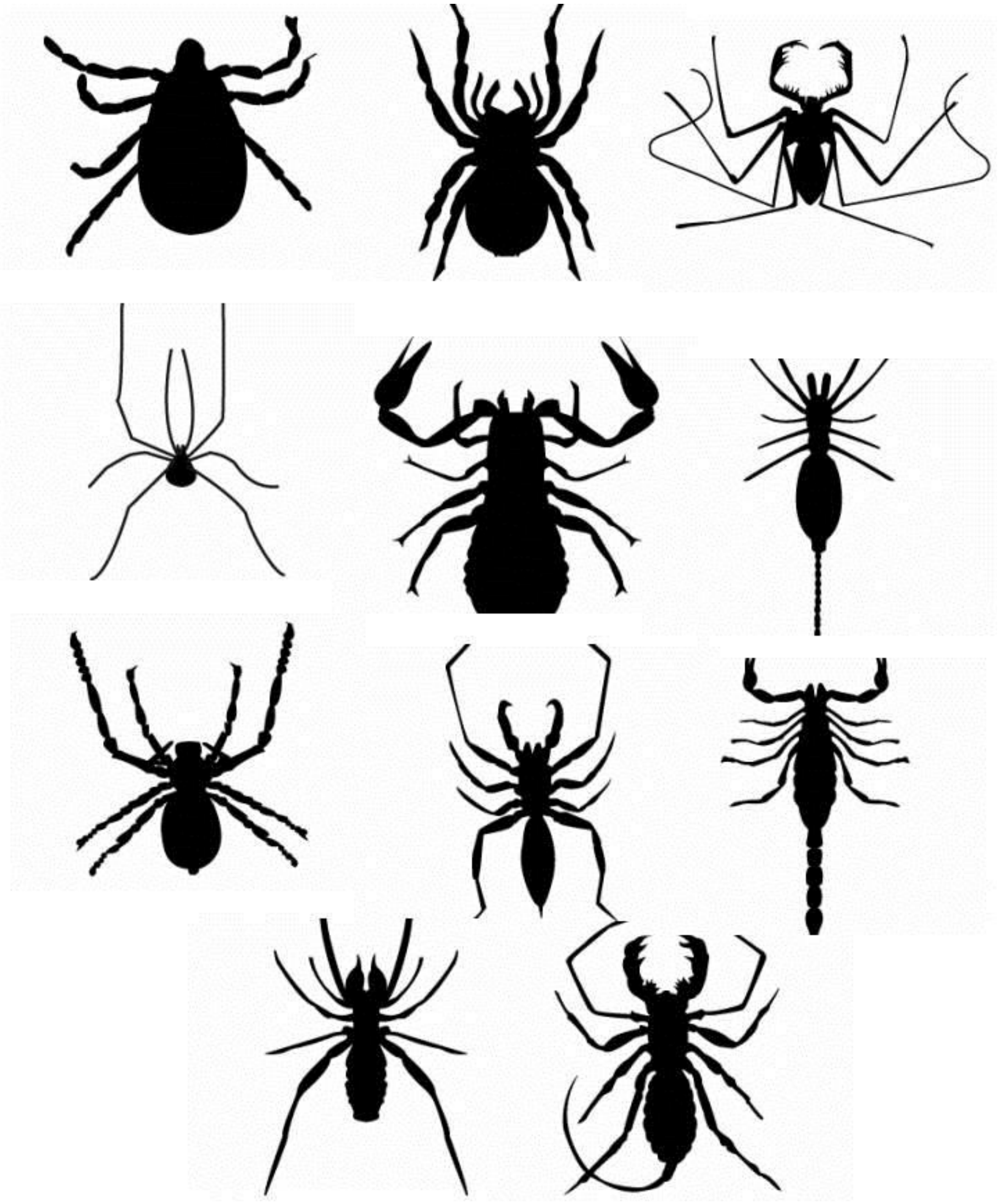

Supplemental Figure 1. Original stamps were used to stamp the *Path of Predators* booklet once participants completed the activities associated with each station. Top Row: Acari, Araneae, Amblypygi; 2nd Row: Opiliones, Pseudoscorpiones, Palpigradi; 3rd Row: Ricinulei, Schizomida, Scorpiones; Bottom Row: Solifugae, Thelyphonida.
