# Supplementary material for: Eight-Legged Encounters—Arachnids, Volunteers, and Art help to Bridge the Gap between Informal and Formal Science Learning
Source: Insects. 2018 Feb 26;9(1):27. doi: 10.3390/insects9010027 (PMC5872292; doi:10.3390/insects9010027)
Supplement: Supplementary file 1 [file insects-09-00027-s001.zip › Eileen Hebets/Supplemental Figure 2.pdf]

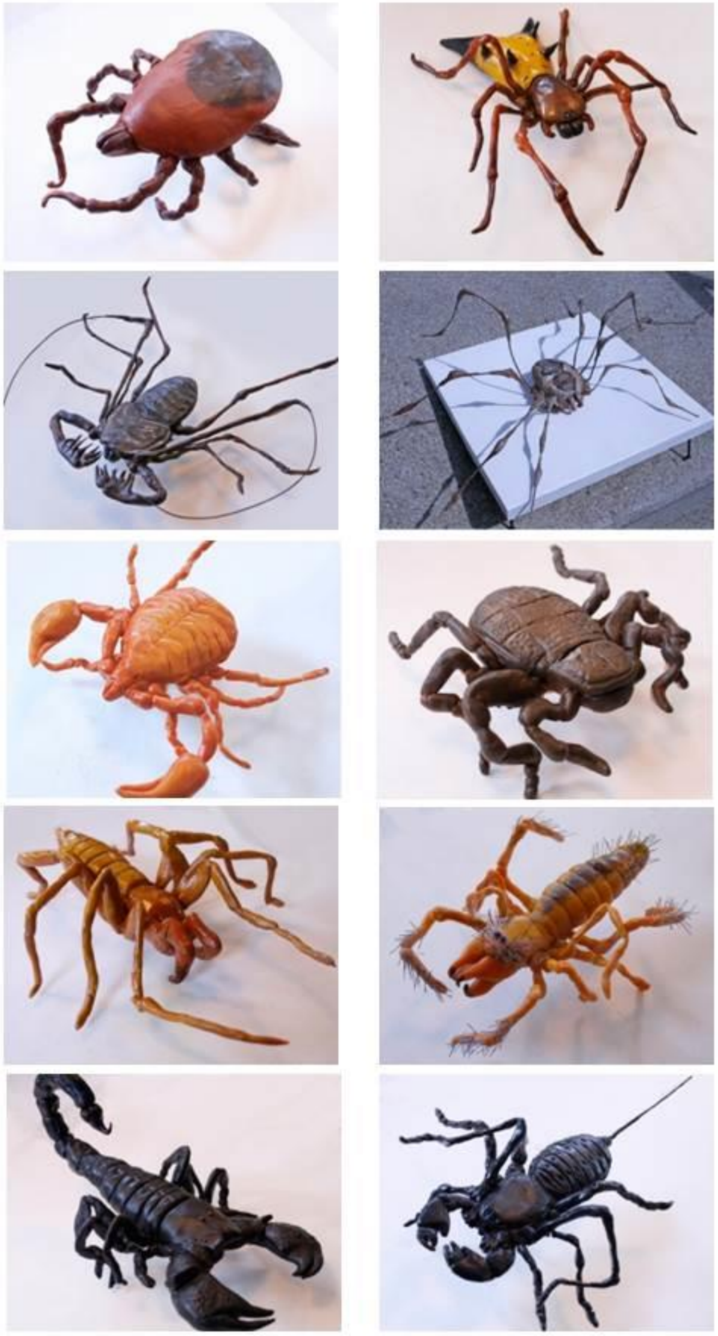

Supplemental Figure 2. Clay sculptures brought to life the diverse arachnids that are the focus of the *Path of Predators*. Top Row: Acari, Araneae; 2nd Row: Amblypygi, Opiliones; 3rd Row: Pseudoscorpiones, Ricinulei; 4th Row: Schizomida, Solifugae; 5th Row: Scorpiones, Thelyphonida.
