# Supplementary material for: Eight-Legged Encounters—Arachnids, Volunteers, and Art help to Bridge the Gap between Informal and Formal Science Learning
Source: Insects. 2018 Feb 26;9(1):27. doi: 10.3390/insects9010027 (PMC5872292; doi:10.3390/insects9010027)
Supplement: Supplementary file 1 [file insects-09-00027-s001.zip › Eileen Hebets/Survey a.pdf]

Eight-Legged Encounters Experience Survey

We would like to learn about your experiences at this event today. All of your responses are anonymous.

1. How interesting did you (and your family) find the following stations:

	Very Interesting	Interesting	Somewhat Interesting	Not Interesting	Does Not Apply/Did Not Visit
a. Myth Busters	○	○	○	○	○
b. Path of Predators	○	○	○	○	○
c. Assemble an Arachnid	○	○	○	○	○
d. Create a Chelicerate	○	○	○	○	○
e. Tissue Paper Flower & Crab Spider	○	○	○	○	○
f. Weave a Web	○	○	○	○	○
g. Microscope Madness	○	○	○	○	○
h. Shining for Spiders	○	○	○	○	○
i. Foraging Experiment	○	○	○	○	○

2. How did you learn about the event?

- ○ Newspaper
- ○ Radio
- ○ School
- ○ Museum Website
- ○ Friends of the Museum
- ○ Facebook
- ○ UNL email
- ○ TV
- ○ Attended other *Sunday with a Scientist*
- ○ Other: ________
- ○ Did not know it was going on

3. In what city and state do you live?

4. How interested are you (and your family) in learning more about science after this event?

- ○ Very Interested
- ○ Interested
- ○ Somewhat Interested
- ○ Not at all Interested

5. (My family) and I learned a lot about science at this event.

- ○ Strongly Agree
- ○ Agree
- ○ Disagree
- ○ Strongly Disagree

6. Please list suggestions for additional topics that may be of interest to you.

7. What did you like about this event?

Thank you for your time!
