# Supplementary material for: Eight-Legged Encounters—Arachnids, Volunteers, and Art help to Bridge the Gap between Informal and Formal Science Learning
Source: Insects. 2018 Feb 26;9(1):27. doi: 10.3390/insects9010027 (PMC5872292; doi:10.3390/insects9010027)
Supplement: Supplementary file 1 [file insects-09-00027-s001.zip › Eileen Hebets/Survey b.pdf]

Eight-Legged Encounters Experience Questionnaire

We would like to learn more about your experiences at this event today. Please take some time to let us know your thoughts.

1. How interesting did you find the following stations/materials?

	Very Interesting	Somewhat Interesting	A Little Interesting	Not Interesting	Did Not Visit
1. Create a chelicerate	○	○	○	○	○
2. Build a burrow	○	○	○	○	○
3. Sticky vs. wooly silk	○	○	○	○	○
4. Weave a web	○	○	○	○	○
5. Microscope madness	○	○	○	○	○
6. Path of predators activity booklet	○	○	○	○	○
7. Community Experiment	○	○	○	○	○

2. After participating in this event....

	Much less likely	Less Likely	The same	More likely	Much more likely
a. how much more or less likely are you to set up your own experiment at home?	○	○	○	○	○
b. how much more or less likely are you to kill a spider in your house?	○	○	○	○	○
c. how much more or less likely are you to attend another similar event?	○	○	○	○	○
d. how much more of less likely are you to take the time to observe a spider, or other arachnid?	○	○	○	○	○
e. how much more or less likely are you to say that you understand what the scientific process is?	○	○	○	○	○
f. how much more or less likely are you to read about arachnids?	○	○	○	○	○

3. After participating in this event, did this make you more or less likely to consider a future job in science?

- ○ Much less likely
- ○ A little less likely
- ○ The same
- ○ A little more likely
- ○ Much more likely
- ○ Not applicable

4. After participating in this event, how much more or less interested are you in learning about scientific discoveries?

- ○ Much less interested
- ○ A little less interested
- ○ The same
- ○ A little more interested
- ○ Much more interested

5. What surprised you about this event?

6. Did you learn anything new?

- ○ No
- ○ Yes

6a. If so, please describe:

7. **What was most meaningful to you from today's exhibit?**

8. **How effective was the artwork in engaging you with the exhibit?**
   - ○ Not at all effective
   - ○ A little effective
   - ○ Somewhat effective
   - ○ Very effective

9. **How effective were the volunteers in engaging you with the exhibit?**
   - ○ Not at all effective
   - ○ A little effective
   - ○ Somewhat effective
   - ○ Very effective

10. **How could the volunteers be more effective in engaging you with the exhibit?**

11. **What did you like best about this event?**

12. **Do you think it is important for these kids of activities to be available to the public?**
    - ○ No
    - ○ Yes → **12a. If so, why?**

13. **What is your gender?**
    - ○ Male
    - ○ Female

14. **What is your current age?**
    - ○ Less than 25
    - ○ 25-34
    - ○ 35-44
    - ○ 45-54
    - ○ 55-64
    - ○ 65 or older

15. **How did you learn about this event?**
    - ○ Newspaper
    - ○ Radio
    - ○ School
    - ○ Museum Website
    - ○ Friends of the Museum
    - ○ Facebook
    - ○ UNL email
    - ○ TV
    - ○ Attended other Sunday with a Scientist
    - ○ Other:
    - ○ Did not know it was going on

16. **What is the zipcode for where you live?**

17. **Please list suggestions for additional topics that may be of interest to you:**

18. **Please use the space below to provide any additional comments or feedback:**
