# Supplementary material for: Eight-Legged Encounters—Arachnids, Volunteers, and Art help to Bridge the Gap between Informal and Formal Science Learning
Source: Insects. 2018 Feb 26;9(1):27. doi: 10.3390/insects9010027 (PMC5872292; doi:10.3390/insects9010027)
Supplement: Supplementary file 1 [file insects-09-00027-s001.zip › Eileen Hebets/Survey e.pdf]

EIGHT-LEGGED ENCOUNTERS (TOLEDO ZOO)

We would like to learn more about your experiences at this event today. Please take some time to let us know your thoughts.

How interesting did you (and your family) find the following stations?	Very Interesting	Somewhat Interesting	A Little Interesting	Not Interesting	Did Not Visit
**1.** Create a chelicerate (clay)	◯	◯	◯	◯	◯
**2.** Path of predators activity booklet	◯	◯	◯	◯	◯

3. After participating in this activity, how much more or less likely are you to kill a spider in your house?

- ◯ Much less likely
- ◯ A little less likely
- ◯ The same
- ◯ A little more likely
- ◯ Much more likely

4. After participating in this activity, how much more or less likely are you to take the time to observe a spider, or other arachnid?

- ◯ Much less likely
- ◯ A little less likely
- ◯ The same
- ◯ A little more likely
- ◯ Much more likely

5. After participating in this activity, how much more or less likely are you to read about arachnids?

- ◯ Much less likely
- ◯ A little less likely
- ◯ The same
- ◯ A little more likely
- ◯ Much more likely

6. After participating in this event, if you are a student, did this make you more or less likely to consider a future job in science?

- ◯ Much less likely
- ◯ A little less likely
- ◯ The same
- ◯ A little more likely
- ◯ Much more likely
- ◯ Not Applicable

7. How much more or less interested are you in learning about scientific discoveries?

- ◯ Much less interested
- ◯ A little less interested
- ◯ The same
- ◯ A little more interested
- ◯ Much more interested

8. How effective was the artwork in engaging you with the exhibit?

- ◯ Not at all effective
- ◯ A little effective
- ◯ Somewhat effective
- ◯ Very effective

9. How effective were the volunteers in engaging you with the exhibit?

- ◯ Not at all effective
- ◯ A little effective
- ◯ Somewhat effective
- ◯ Very effective

How could the volunteers be more effective in engaging you with the exhibit?

Did you learn anything new? If so, please briefly describe.

Do you think it is important for these kinds of activities to be available to the public? If so, why?

Please use the space below to provide any additional comments or feedback:

Thank you!
