# Supplementary material for: Eight-Legged Encounters—Arachnids, Volunteers, and Art help to Bridge the Gap between Informal and Formal Science Learning
Source: Insects. 2018 Feb 26;9(1):27. doi: 10.3390/insects9010027 (PMC5872292; doi:10.3390/insects9010027)
Supplement: Supplementary file 1 [file insects-09-00027-s001.zip › Eileen Hebets/Survey f.pdf]

Morrill Hall 2014

Youth Survey

How much did you like the...	A lot	A little	Not much or not at all	Did Not Visit
1. Create a chelicerate	○	○	○	○
2. Build a burrow	○	○	○	○
3. Sticky vs. wooly silk	○	○	○	○
4. Weave a web	○	○	○	○
5. Microscope madness	○	○	○	○
6. Path of predators activity booklet	○	○	○	○
7. Community Experiment	○	○	○	○

Do you want to learn more about spiders?

- ○ Yes
- ○ No

Do you want to learn more about science?

- ○ Yes
- ○ No

Do you want to do your own research at home?

- ○ Yes
- ○ No

When you grow up, do you want a job in science?

- ○ Yes
- ○ No

How old are you?

(INTERVIEWER MAKE NOTE OF THIS) What is your gender?

- ○ Male
- ○ Female
