# Supplementary material for: Eight-Legged Encounters—Arachnids, Volunteers, and Art help to Bridge the Gap between Informal and Formal Science Learning
Source: Insects. 2018 Feb 26;9(1):27. doi: 10.3390/insects9010027 (PMC5872292; doi:10.3390/insects9010027)
Supplement: Supplementary file 1 [file insects-09-00027-s001.zip › Eileen Hebets/Volunteer Survey.pdf]

Eight-Legged Encounters (Denver Museum of Science and Nature)
Volunteer Experience Questionnaire

We would like to learn more about your experiences at this event today. Please take some time to let us know your thoughts. Feel free to use the back of the survey for any additional comments or suggestions.

1. After participating in this event….

	Much less likely	Less Likely	The same	More likely	Much more likely
**a.** how much more or less likely are you to volunteer at another outreach event?	◯	◯	◯	◯	◯
**b.** how much more or less likely are you to go into a science career?	◯	◯	◯	◯	◯
**c.** how much more or less likely are you to go into an education career?	◯	◯	◯	◯	◯

2. After this event, how much more or less interested are you in conveying science to the general public?

- ◯ Much less interested
- ◯ A little less interested
- ◯ The same
- ◯ A little more interested
- ◯ Much more interested

3. After this event, do you think it is easier or harder than you thought before to convey science to a general audience?

- ◯ Easier
- ◯ The same
- ◯ Harder

4. In what ways could you experience with this event be improved?

5. What was your favorite part of the experience?

6. Did you learn anything at this event?

- ◯ No
- ◯ Yes → **What did you learn?**

7. What surprised you about this event?

8. Rate your overall experience volunteering at this event?

- ◯ 1 (the worst)
- ◯ 2
- ◯ 3
- ◯ 4
- ◯ 5 (the best)

9. What station did you work at?

10. Were you provided with sufficient information to volunteer at this station?

- ◯ Yes
- ◯ No → **What additional information would have been helpful?**

11. What is your gender?

- ◯ Male
- ◯ Female

12. What is your current age?

- ◯ Less than 25
- ◯ 25-34
- ◯ 35-44
- ◯ 45-54
- ◯ 55-64
- ◯ 65 or older

a. What races or races do you consider yourself to be(select all that apply)?

- ☐ White (Caucasian)
- ☐ Black or African American
- ☐ Asian
- ☐ American Indian or Alaska Native
- ☐ Native Hawaiian or other Pacific Islander
- ☐ Other, specify:
